# Role of VEGFs/VEGFR-1 Signaling and Its Inhibition in Modulating Tumor Invasion: Experimental Evidence in Different Metastatic Cancer Models

**DOI:** 10.3390/ijms21041388

**Published:** 2020-02-18

**Authors:** Claudia Ceci, Maria Grazia Atzori, Pedro Miguel Lacal, Grazia Graziani

**Affiliations:** 1 Department of Systems Medicine, University of Rome Tor Vergata, Via Montpellier 1, 00133 Rome, Italy; claudiaceci@hotmail.it (C.C.); mariagraziaatzori.2@gmail.com (M.G.A.); 2 Laboratory of Molecular Oncology, “Istituto Dermopatico dell’Immacolata-Istituto di Ricovero e Cura a Carattere Scientifico”, IDI-IRCCS, Via dei Monti di Creta 104, 00167 Rome, Italy; p.lacal@idi.it

**Keywords:** angiogenesis, VEGFR-1, Flt-1, PlGF, VEGF-A, cancer, metastasis, immune escape, melanoma

## Abstract

The vascular endothelial growth factor (VEGF) family members, VEGF-A, placenta growth factor (PlGF), and to a lesser extent VEGF-B, play an essential role in tumor-associated angiogenesis, tissue infiltration, and metastasis formation. Although VEGF-A can activate both VEGFR-1 and VEGFR-2 membrane receptors, PlGF and VEGF-B exclusively interact with VEGFR-1. Differently from VEGFR-2, which is involved both in physiological and pathological angiogenesis, in the adult VEGFR-1 is required only for pathological angiogenesis. Besides this role in tumor endothelium, ligand-mediated stimulation of VEGFR-1 expressed in tumor cells may directly induce cell chemotaxis and extracellular matrix invasion. Furthermore, VEGFR-1 activation in myeloid progenitors and tumor-associated macrophages favors cancer immune escape through the release of immunosuppressive cytokines. These properties have prompted a number of preclinical and clinical studies to analyze VEGFR-1 involvement in the metastatic process. The aim of the present review is to highlight the contribution of VEGFs/VEGFR-1 signaling in the progression of different tumor types and to provide an overview of the therapeutic approaches targeting VEGFR-1 currently under investigation.

## 1. Introduction

The evolution of numerous types of cancer is associated with the transition to the invasive phase, with the so-called angiogenic switch and metastatic spreading. The process of formation and growth of new blood vessels from pre-existing vessels implies complex communications between tumor cells, endothelial cells and extracellular matrix (ECM) components and secretion of a variety of growth factors. Among the proteins secreted by cancer cells or by cells of the tumor microenvironment that stimulate blood vessel formation, the vascular endothelial growth factors (VEGFs) play a key role in several tumors [1,2,3].

Members of the human VEGF family comprise VEGF-A, -B, -C, -D, and placenta growth factor (PlGF). In particular, VEGF-A, the first angiogenic factor identified, is critical not only for pathological angiogenesis and consequent tumor cell dissemination, but also for physiological angiogenic processes, such as embryonic vascular development, skeletal morphogenesis and growth, post-natal angiogenesis, as well as tissue repair and reproductive functions in the adult. Mice lacking even a single *VEGF-A* allele exhibit impaired development of early vasculature and die at E11-E12 [4]. PlGF, the second discovered member of the VEGF family, named after its cloning from a human placental cDNA library [5,6], is instead dispensable for normal development and physiological angiogenesis processes. Indeed, *PlGF*-deficient mice do not show phenotypic abnormalities; conversely, PlGF is involved in several diseases associated with pathological angiogenesis, including cancer [7,8]. VEGF-B has been involved in inflammatory angiogenesis [9] since knockout mice exhibit reduced inflammation and angiogenesis in collagen-induced models of arthritis. Differently from VEGF-A and VEGF-B [10,11], for which several isoforms are produced through alternative mRNA splicing, the various VEGF-C and VEGF-D forms derive from the proteolytic cleavage of their precursors [12,13]. VEGF-C is highly expressed during the embryo development in regions where lymphatic vessels are formed and decreases with age in most tissues, except in the lymph nodes [14]. Consistently, mice lacking both *VEGF-C* alleles do not develop lymphatic vessels and embryos die for tissue edema [15,16]. VEGF-D is mainly expressed in the lung and the skin during embryogenesis and plays a role in angiogenesis as well as in lymphangiogenesis [16]. In tumors, VEGF-D promotes the growth of lymphatic vessels and lymphatic metastasis [17].

**Figure 1:**
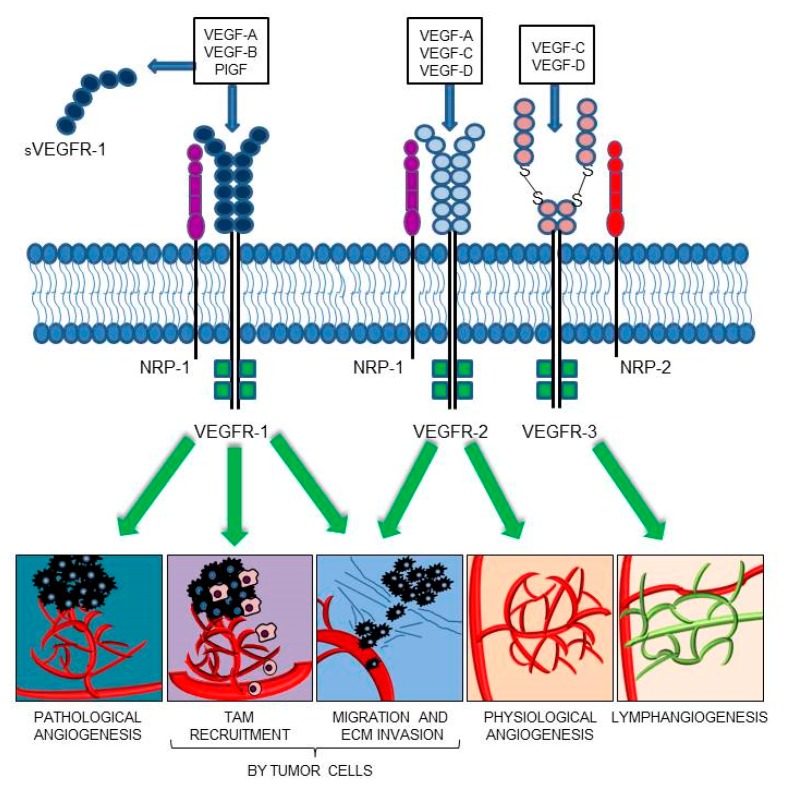
VEGF family members and their receptors. VEGF-A proangiogenic signaling is mediated via interaction with VEGFR-2 or VEGFR-1. The soluble VEGFR-1 form (sVEGFR-1) functions as a decoy receptor, preventing membrane receptor activation. VEGF-B and PlGF only bind to VEGFR-1, playing a key role in pathological angiogenesis and inflammation. Furthermore, VEGFR-1 activation contributes to the recruitment of tumor-associated macrophages (TAMs) and cancer immune escape. VEGFR-1 and VEGFR-2 activation in tumor cells directly stimulates migration and extracellular matrix (ECM) invasion. VEGF-C and VEGF-D mainly activate VEGFR-3, which is required for developmental and pathological lymphangiogenesis. The VEGF-E, a selective VEGFR-2 ligand, and VEGF-F, a VEGFR-1 and VEGFR-2 ligand, have been omitted from the drawing; VEGF-E is a VEGF homolog of viral origin and VEGF-F is a snake venom VEGF.

The members of the VEGF family exert their functions by binding and activating membrane receptors that exhibit tyrosine-kinase activity (RTKs), including vascular endothelial growth factor receptor 1 (VEGFR-1/Flt-1), VEGFR-2 (KDR/Flk-1), and VEGFR-3 (Flt-4) [18,19] (Figure 1). All VEGFRs contain seven immunoglobulin (Ig) homology domains, which comprise the ligand-binding site, and an intracellular region endowed with tyrosine kinase (TK) activity, which transduces the signal. In blood vascular endothelial cells, VEGF-A signaling is mainly mediated by the activation of VEGFR-2 [20]. The VEGF-A also interacts with VEGFR-1; conversely, PlGF and VEGF-B exclusively bind to VEGFR-1 [21,22]. Due to its relatively weak kinase activity, VEGFR-1 was initially considered an inhibitory receptor of VEGF-A, which prevented its binding to VEGFR-2. However, PlGF/VEGFR-1 and VEGF-A/VEGFR-1 signaling pathways were later found to be responsible for the neovessel formation associated with a variety of pathologies, including cancer [23,24,25]. The VEGFR-1 is also secreted in the ECM as a soluble isoform (sVEGFR-1), which derives from alternative splicing of the *VEGFR-1* mRNA [26]. The sVEGFR-1 comprises the ligand-binding domain of the membrane protein and acts as a decoy receptor of VEGF-A, VEGF-B, and PlGF, due to its ability to sequester these ligands. Moreover, the sVEGFR-1 can interact with VEGFR-2, thus blocking its activity. Therefore, the sVEGFR-1 exerts antiangiogenic, anti-edema, and anti-inflammatory activities, and its dysregulation has been associated with different pathological processes. For example, the expression of sVEGFR-1 by epithelial cells contributes to the corneal avascularity and its transfection in lacrimal glands has been shown to prevent the pathological corneal neovascularization [27,28]; the pathogenesis of pre-eclampsia, typically occurring in the last trimester of pregnancy, has been related to sVEGFR-1 production by placenta and subsequent neutralization of VEGF-A and PlGF signaling [29,30]; a low sVEGFR-1 to VEGF-A ratio has been correlated with higher tumor malignancy/invasiveness and poor patients’ survival [31,32,33,34,35,36,37]. The sVEGFR-1 may also play a proangiogenic and protumoral action by activation of β1 integrin, which results in stimulation of endothelial cell adhesion and chemotaxis [38,39,40].

By contrast, VEGF-C and VEGF-D activate VEGFR-3, a receptor endowed with an important role both in physiological and pathological lymphangiogenesis, and are involved in tumor progression [16,41]. In solid tumors, activation of the VEGF-C/VEGFR-3 or VEGF-D/VEGFR-3 pathways in lymphatic endothelial cells participates in tumor spreading, thanks to the formation of new lymphatic vessels around and within the tumor mass [41,42]. In hematological malignancies, the VEGF-C/VEGFR-3 axis promotes cancer cell proliferation and resistance to chemotherapy [42]. VEGF-D can also induce dilation of collecting lymphatic vessels, which favors the transport of tumor cells through the lymphatic network, by a mechanism requiring prostaglandin synthesis [43]. Moreover, both VEGF-C and VEGF-D may also promote angiogenesis due to activation of VEGFR-2.

Ligand binding induces changes in the VEGFRs transmembrane domain conformation, thereby stimulating receptor homodimerization, activation, and autophosphorylation [44]. Not surprisingly, recently developed antibodies, aimed at therapeutically modulating VEGFRs activity, block their homotypic interactions, and hence their activation [45,46].

Several co-receptors that modulate the VEGFR signaling have also been identified. The semaphorin receptor neuropilin-1 (NRP-1) is an example of VEGFR-1/-2 co-receptor that regulates VEGF-A-mediated endothelial permeability by regulating receptor phosphorylation and downstream signaling [47,48,49], whereas NRP-2 is a co-receptor for VEGFR-3, participating in the development and organization of the lymphatic vascular network [50] (Figure 1). Even in the absence of RTK family members, NRP-1 is able to respond to different signals, among which PlGF, and to activate downstream signaling pathways which eventually promote cell survival, proliferation, motility, and angiogenesis, both in non-malignant and malignant contexts [51,52,53,54,55].

## 2. Role of VEGFR-1 and its Ligands in Tumor Progression

The VEGF/VEGFR signaling pathway is upregulated in many types of cancers, contributing to uncontrolled angiogenesis and metastatic spreading. As the tumor mass increases in size and the oxygen availability decreases, the tumor itself or stromal cells of the tumor microenvironment produce proangiogenic factors, which induce endothelial cells to proliferate and migrate. This results in the formation of new vessels that deliver oxygen and nutrients to tumor cells. Not surprisingly, the hypoxia-inducible factor-α (HIF-1α), a transcription factor specifically activated when oxygen levels decrease, controls *VEGF-A* gene transcription. The new blood vessels also provide an excellent route through which tumor cells can generate metastases. However, the new vasculature originated by tumor-released VEGF-A is usually structurally and functionally abnormal [56,57,58]. The excessive production of VEGF-A by hypoxic tumor cells is responsible for the generation of chaotically organized tumor vessels, with an irregular and tortuous appearance, instead of the hierarchical structural organization normally found in non-cancerous vascular networks [59]. Besides hypoxia, inflammation is another condition that contributes to cancer progression via VEGFs/VEGFRs signaling. Activated lymphocytes and other cells of the immune compartment infiltrating the tumor may release VEGF-A and inflammatory cytokines able to increase HIF-1α and consequently VEGF-A synthesis [60,61,62,63].

As expected, VEGF-A overexpression frequently correlates not only with enhanced cancer invasiveness, but also with a high risk of tumor recurrence and unfavorable prognosis [64]. On this basis, inhibition of the VEGF-A/VEGFRs signaling represents a widely used approach for cancer treatment through the use of the anti-VEGF-A and anti-VEGFR-2 monoclonal antibodies (mAbs) bevacizumab and ramucirumab, respectively; the chimeric molecule ziv-aflibercept; or a number of multi-targeted small-molecule TK inhibitors [65,66]. Unfortunately, agents that hamper VEGF-A/VEGFR-2 signal transduction also inhibit physiological angiogenesis and produce severe systemic adverse effects. Conversely, the selective targeting of VEGFR-1 signal transduction or VEGFR-1 exclusive ligands (i.e., PlGF or VEGF-B) might represent a suitable approach to impair tumor-associated vessel formation. In fact, PlGF and VEGFR-1 are overexpressed in several tumor types and contribute to ECM invasion and resistance to anti-VEGF-A therapies. Importantly, as VEGFR-1 plays a critical role in tumor-associated angiogenesis but not in physiological angiogenesis, VEGFR-1-blocking therapies are expected to cause less adverse effects than molecules targeting VEGFR-2 [66,67]. The levels of sVEGFR-1 also seem to play a significant role in cancer progression: in several cancer types, the VEGF-A/sVEGFR-1 ratio detected in tumor tissue, serum or plasma samples, has been shown to correlate with disease aggressiveness, malignancy grade, survival and response to the therapy [31,32,33,34,35]. In regard to VEGF-B, ectopic expression of the growth factor in pancreatic β-cells of transgenic mice prevented the formation of neuroendocrine tumors likely displacing VEGF-A or PlGF from VEGFR-1 [68]. More recently, VEGF-B was found to significantly induce remodeling of the tumor microvasculature, which creates highly permissive conditions for tumor cell invasion and metastasis, in a VEGF-A independent manner [69]. Overall, its role on tumor progression is less well-established than that of VEGF-A or PlGF and data on its involvement in cancer aggressiveness and metastasis have been reported only in certain cancer types (see below).

Inhibition of angiogenesis and metastasis formation is not the only effect deriving from the blockade of VEGF-A or PlGF signaling. Actually, several reports have demonstrated that, in addition to the promotion of angiogenesis and tumor invasive behavior, VEGF-A and PlGF are endowed with immunosuppressive properties, as they affect the function of immune cells [70,71]. Therefore, by inhibiting VEGF-A or PlGF signaling, it is possible to reduce the risk of tumor immune escape. High plasma levels of VEGF-A in cancer patients were found to correlate with a reduced maturation of dendritic cells (DCs), causing impaired differentiation of effector T and natural killer (NK) cells [72]. In particular, VEGF-A, via the VEGFR-1 signaling, prevented the activation of the transcription factor nuclear factor-κB (NF-κB) and the normal DCs differentiation. Through the activation of VEGFR-1, VEGF-A and PlGF are also able to recruit monocytes/macrophages and to promote the development of tumor-associated macrophages (TAMs), an immune cell population which contributes to tumor-induced inflammation, growth, invasion, and metastases [73,74,75,76,77,78].

In the following sections, we summarize the experimental evidence supporting a role for VEGFR-1 and its ligands on the progression and metastatic potential of different tumor types.

### 2.1. Lung Cancer

Lung cancer is the most frequent cancer and the leading cause of cancer-related deaths. Eighty-five percent of total lung malignancies are represented by non-small-cell lung cancer (NSCLC), a difficult-to-treat disease, due to the high frequency of metastasis formation. By lymphatic as well as blood vessels, NSCLC mainly metastasizes in the bone. The brain, liver, and adrenal glands represent other preferential metastatic sites [79,80]. Tumor-associated angiogenesis has been implicated in NSCLC progression and the role of VEGF-A in this context has been extensively reviewed elsewhere [81]. The anti-VEGF-A bevacizumab is the standard regimen for advanced/metastatic non-squamous NSCLC in the first-line setting, in combination with platinum-based chemotherapy [82]. Interestingly, patients with higher expression levels of VEGF-A, VEGFR-1, and VEGFR-2 in the tumor showed a markedly shorter survival time, compared with patients exhibiting low VEGF-A levels or only expressing one receptor type. Therefore, these three factors were proposed as prognostic markers for NSCLC cancer patients [83].

NSCLC is often associated with a hypoxic environment leading to HIF-1α overexpression [84], and HIF-1α knockdown in the NCI-H157 lung carcinoma cell line has been shown to reduce VEGF-A expression and cell invasiveness [85]. Several microRNAs (miRNAs) were also reported to modulate VEGF-A expression and to be downregulated in lung cancer cell lines. In particular, miR-126, miR-497, MiR-206, miR-29c, miR-135a, and miR-195 expression inversely correlated with VEGF-A production in different lung cancer cell lines or in patients’ tumor samples [86,87,88,89,90,91]. Moreover, interleukin-17 (IL-17) was found to stimulate NSCLC-associated angiogenesis by augmenting the secretion of various angiogenic factors. In particular, IL-17 seemed to induce *VEGF-A* gene transcription by activating the signal transducer and activator of transcription 3 (STAT3)/Gα-Interacting Vesicle-associated protein (GIV) signaling pathway [92]. Furthermore, an autocrine loop has been identified in NSCLC, in which tumor-derived VEGF-A induces the secretion of VEGF-A itself and other proangiogenic factors, an effect mediated by the phosphatidylinositol-3-kinase (PI3K)/Protein Kinase B (AKT), RAS/extracellular signal-regulated kinase (ERK), and STAT3 signaling pathways [81]. The VEGF-A165 was found to regulate the expression of the sVEGFR1-i13 splice variant of sVEGFR-1 in squamous lung carcinoma cells through (sex-determining region Y (SRY)-Box2) (SOX2) and serine-arginine-rich splicing factor 2 (SRSF2) proteins [93]. The sVEGFR-1-i13 splice variant was reported to increase during treatment with antiangiogenic therapies and to contribute to the progression of squamous lung carcinoma [93]. In fact, besides acting as inhibitor of angiogenesis, this sVEGFR-1 variant is a component of the ECM that binds to the α5β1 integrin and stimulates the adhesion and migration of endothelial cells [38,94].

Besides VEGF-A, also PlGF resulted to be overexpressed in NSCLC specimens when compared with paired non-cancer tissues. In particular, higher PlGF expression was detected in tumor samples collected from patients with distal metastases compared to patients lacking metastases and was associated with poor survival. Moreover, overexpression of PlGF was found to correlate with increased cell invasiveness and enhanced expression of matrix metalloproteinase 9 (MMP9), which induces ECM degradation. Consistently, inhibition of PlGF by a small short hairpin interfering RNA decreased the levels of MMP9 and tumor invasiveness. Suppression of mitogen-activated protein kinases (MAPK)-p38 levels abolished the effect of PlGF on MMP9 expression, thus identifying the signaling pathway involved in such effect [95]. High PlGF levels were also associated with induction of the splicing regulatory factor SRp40 and with an increased ratio between the proangiogenic (VEGF-A165) and the antiangiogenic (VEGF-A165b) isoforms of VEGF-A. These data suggest that PlGF might increase NSCLC metastases through SRp40-mediated splicing of the *VEGF-A* mRNA [96]. In a transwell-based co-culture model, PlGF secreted by NSCLC cells also induced macrophage polarization to TAM, by stimulating the membrane VEGFR-1 expressed by these cells, which in turn favors the growth and invasive potential of the tumor. Therefore, the cross-talk between TAMs and NSCLC cells via PlGF/VEGFR-1 interaction is another mechanism responsible for disease progression [78]. Furthermore, the VEGFR-1 relevance to NSCLC aggressiveness was confirmed by the observation that patients with squamous cell carcinoma and high VEGF-B expression showed poorer survival compared to those with low VEGF-B expression [69].

Small-cell lung cancer (SCLC) accounts for approximately 15% of all lung cancer cases and mainly metastasizes to the liver [80,97]. Like NSCLC, SCLC is also characterized by the overexpression of several VEGF family members and poor outcomes. In particular, VEGF-A levels have been demonstrated to directly correlate with the microvessel density. Consistently, the combination of angiogenesis inhibitors, like bevacizumab or ziv-aflibercept, with traditional chemotherapy, significantly improved the overall response rate (ORR) and progression-free survival (PFS) of patients with SCLC [98].

Malignant pleural mesothelioma is an extremely aggressive tumor, which easily spreads locally to the nearby structures as well to distant sites [99]. A semiquantitative analysis of PlGF, VEGFR-1, NRP-1, and NRP-2 levels in this tumor, performed by immunohistochemistry on tumor specimens from patients undergoing extrapleural pneumonectomy, showed that the four factors were specifically overexpressed in mesothelioma [100]. Similar results were obtained by western blot and immunohistochemistry analyses of malignant mesothelioma cell lines and tissue samples, respectively. Cell treatment with an anti-human PlGF antibody decreased mesothelioma cell survival [101]. Furthermore, PlGF expression inversely correlated with patient survival, but it did not correlate with the tumor stage [100].

Finally, the lung is often the site of spreading of several other metastatic cancers. Bone marrow-derived cells (BMDCs), which are known to express VEGFR-1, are mobilized in response to cytokines produced by the primary tumor and form “pre-metastatic niches” in the lung, even before the arrival of cancer cells [102]. Blockade of VEGFR-1 activity in tumor-bearing mice (subcutaneously injected with murine Lewis lung carcinoma or B16 melanoma cells) by intraperitoneal treatment with the anti-murine VEGFR-1 mAb MF-1, did not affect the number of BMDCs in the pre-metastatic lung. The same result was obtained in mice exhibiting a genetic deletion of the *VEGFR-1* TK domain. Nevertheless, after metastatic nodule formation, VEGFR-1 blockade led to a decrease of BMDCs infiltration inside and around the metastatic nodules. Thus, VEGFR-1 activity is dispensable for the formation of metastatic tumor nodules in the pre-metastatic lungs but is required for the subsequent BMDCs infiltration that is essential for the growth of metastatic nodules [103].

### 2.2. Liver Cancer

Primary liver cancer includes hepatocellualar carcinoma and cholangiocarcinoma, which derive from hepatocytes and the intrahepatic bile duct epithelium, respectively. These tumors represent a leading cause of cancer-related deaths worldwide.

Hepatocellular carcinoma represents 80% of primary liver cancer, which is the sixth most frequent type of cancer. Among extrahepatic metastatic sites, the lung is commonly involved, followed by lymph nodes, bone, and adrenal glands [104]. Hepatocellular carcinoma is a hypervascularized cancer type, and dysregulation of several angiogenic pathways, including those activated by the VEGF family members, has been involved in the development and progression of this tumor [105]. In fact, in patients with hepatocellular carcinoma high circulating VEGF-A levels have been reported that correlated with tumor angiogenesis and reduced survival and were considered independent predictors of survival [106,107,108,109]. Moreover, the VEGF-B186 isoform resulted to be more frequently upregulated compared to the VEGF-B167 one and its expression correlated with tumor growth and invasiveness [110]. Low expression of the miR-199a-3p reported in hepatocellular carcinoma and other tumor types has been suggested to contribute to angiogenesis and disease progression. In fact, miR-199a-3p was found to reduce the VEGF-A secretion by cancer cells and the expression of VEGFR-1 and VEGFR-2 on endothelial cells [111]. Restoration of miR-199a-3p in human hepatocellular carcinoma cells reduced their in vivo tumorigenic and metastatic potential [111].

The involvement of PlGF in liver tumorigenesis has been demonstrated in chemically-induced and transgenic mouse models of hepatocellular carcinoma by PlGF silencing or pharmacological inhibition using the murine anti-PlGF 5D11D4 mAb [112,113]. PlGF blockade resulted in normalization of tumor-associated vessels, reduced tumor nodule formation in the liver, and increased animal survival. Similar findings were obtained in chemically-induced hepatocellular and cholangiocarcinoma in vivo models, where treatment with the 5D11D4 mAb decreased tumor burden and infiltration by protumoral M2 cells [114].

Furthermore, VEGFR-1 activation by VEGF-B caused the typical molecular and morphological alterations of the epithelial/mesenchymal transition (EMT), which facilitated cell migration and ECM invasion. Blockade of the receptor by the neutralizing anti-VEGFR-1 IMC-18F1 mAb counteracted EMT induction [115]. A dual mechanism has been identified through which VEGF-B-induced activation of VEGFR-1 enhances cell migration and invasion: stimulation of MMP9 signaling and increased expression of the EMT zinc-finger regulator Snail. Furthermore, as patients with the worst clinical outcome showed higher expression of VEGFR-1 or coexpression of VEGFR-1 and MMP9 in the tumor than in peritumoral tissues, VEGFR-1 has been proposed as a prognostic marker for hepatocellular carcinoma [116,117,118].

In human cholangiocarcinoma, VEGF-A expression was associated with angiogenesis, metastasis and tumor recurrence [119]. High VEGF-A levels in human cholangiocarcinoma tissues have been correlated to a marked decrease of miR-101, a miRNA that directly targets VEGF-A mRNA and represses VEGF-A gene transcription by inhibiting cyclooxygenase-2 (COX-2) and prostaglandin E [120]. The antiangiogenic/tumor suppressive activity of miR-101 was confirmed by in vivo experiments in xenograft models of cholangiocarcinoma showing that overexpression of this miRNA in CCLP1 and HuCCT1 cell lines markedly inhibited tumor growth and progression [120].

High VEGF-A expression was also detected in gallbladder carcinoma, the most common malignancy of the biliary tract, where it correlated with histological tumor grade and TNM (tumor, node, metastasis) staging [121,122]. In this tumor type, PlGF was also overexpressed and stimulated EMT through c-MYC upregulation and consequent induction of miR-19a, a miRNA which plays an important role in gallbladder metastasis and stemness. Consistently, patients with high PlGF and miR-19a levels in the tumor showed a shorter overall survival (OS) than patients with low expression [123].

Liver is also the most frequent site of metastases from solid tumors. VEGFR-1 has been reported to be required for the vascularization of liver metastases from renal cell carcinoma (RCC), whereas it was dispensable for lung metastases [124]. In fact, blockade of VEGFR-1 by the MF-1 mAb in syngeneic murine RCC models induced 31% reduction in the growth of liver metastases, whereas blockade of VEGFR-2 had minimal effects. In the case of metastases from colon carcinoma, only the neutralization of both VEGFR-1 and VEGFR-2 was able to decrease the size of liver metastasis [124]. A possible mechanism responsible for the more prominent role of VEGFR-1 in liver metastasis formation compared to VEGFR-2 could be the different receptor ability to stimulate STAT3 signaling. Indeed, VEGR-1 activation by PlGF stimulated this pathway more in liver endothelial than in lung endothelial cells, whereas selective VEGFR-2 activation had the opposite effect [124]. In this regard, note that STAT3 signaling has been identified as one of the molecular mechanisms involved in the establishment of pro-metastatic niches in the liver [125]. Furthermore, it has been hypothesized that the recruitment of VEGFR-1 positive BMDCs might differ between lung and liver metastases.

### 2.3. Kidney Cancer

Kidney cancer accounts for 4% of all cancer types occurring in the adults and is the 7th and 10th most common cancer in men and women, respectively [126]. Approximately ninety percent of cases are represented by RCC, one of the most common metastatic tumors. RCC usually metastasizes to the lung, bone, lymph nodes, liver, adrenal gland, and brain [127]. Clear-cell renal cell carcinoma (ccRCC) is the most common histological type and the most aggressive and death-inducing form of RCC, with metastasis occurring in one third of newly diagnosed cases [127].

Angiogenesis plays a pivotal role in the development and progression of kidney cancer. The ccRCC is characterized by mutations or epigenetic inactivation of the *von Hippel–Lindau* (*VHL*) tumor suppressor gene, which are considered to play a key role in VEGF-A overexpression. The *VHL* gene encodes for the E3 ubiquitin ligase that interacts with HIF-1α under oxygenated conditions, targeting it for polyubiquitination and proteasomal degradation (seminal discovery of the 2019 Nobel laureates in Physiology or Medicine William G. Kaelin Jr. and Peter J. Ratcliffe) [128,129]. In hypoxic conditions, HIF-1α does not bind to VHL protein and is not degraded. Thus, in the presence of low expression or dysfunctional VHL, the HIF-1α accumulates and activates a number of hypoxia-driven genes, including *VEGF-A*.

Several single nucleotide polymorphisms identified in the *VEGF* gene have been reported to be associated with RCC risk, tumor growth, and metastases [130,131,132]. In particular, two meta-analysis studies indicated that the *VEGF* -2578C/A, +936C/T, and +405G/C polymorphisms correlated with elevated risk of RCC, especially in the Asian populations [133,134].

Among the molecular mechanisms that regulate VEGF-A levels, the endoribonuclease Dicer, which cleaves pre-miRNAs into mature miRNAs, was recently reported to inhibit the expression of this angiogenic factor. In ccRCC, Dicer was significantly decreased compared to normal tissues and its downregulation has been associated to poor prognosis and metastasis formation [135]. This effect has been attributed to the ability of Dicer to significantly suppress the expression of VEGF-A and MMP2, as demonstrated by in vitro and in vivo preclinical studies [135].

Compared to healthy renal tissues, RCC was found to express higher levels not only of VEGF-A but also of VEGFR-1. Conversely, no difference in VEGFR-2 expression was detected between epithelial or stromal compartments of tumoral and non-tumoral kidney sites. VEGF-A protein levels were associated with tumor size, tumor grade, and metastasis at diagnosis [136]. Another study demonstrated a concomitant higher expression of *VEGF-B* and *VEGFR-1* mRNA in RCC tissues compared with normal kidney tissues, whereas no variation was detected in *VEGF-C* and *VEGFR-3* [137].

Tumor infiltration by BMDC expressing VEGFR-1 may contribute to the neovessel formation and immune escape in RCC [138]. In fact, orthotopic inoculation of the human RCC Caki-1 cell line into nude mice induced an increase of VEGFR-1+/CD11b+ myeloid cells in the peripheral blood, which was abrogated by the administration of the anti-VEGF-A mAb bevacizumab. VEGFR-1 positive cells were also detected in the peripheral blood and tumor tissues of patients with metastatic RCC [138]. The induction of VEGFR-1 expression in myeloid cells was also observed in vitro by exposing bone marrow cells or myeloid cells to tumor-conditioned medium or oxidative stress, respectively. Induction of oxidative stress in myeloid cells resulted in the acquisition of an immunosuppressive phenotype, as demonstrated by the ability of these cells to inhibit T lymphocyte proliferation [138]. Therefore, it can be hypothesized that the increased VEGF-A levels in the serum of patients with metastatic RCC favors the recruitment at the tumor site of VEGFR-1 positive immunosuppressive myeloid cells which contribute to immune escape and neoangiogenesis.

In regard to PlGF, the plasma concentrations of this VEGFR-1 ligand have been reported to increase in patients with RCC after treatment with the multi-targeted RTK inhibitor sunitinib, suggesting a correlation with angiogenic rescue and resistance to antiangiogenic therapies [139]. However, PlGF neutralization by the TB403 mAb did not inhibit the growth of sunitinib-resistant ccRCC xenografts, which did not express VEGFR-1 [140].

### 2.4. Glioblastoma

Glioblastoma is the most aggressive primary malignant brain tumor, with a median survival of approximately 10 months [141]. It can be classified as primary (de novo) or secondary when glioblastoma derives from low-grade tumors [142]. Given the short survival of patients, there is not enough time to develop metastases. Although extracranial metastases are rare, most documented cases involve leptomeningeal spreading to the spinal cord; but metastases to the liver, skin, spleen, lung, peritoneum, and lymph nodes may also occur [143].

Studies performed in patients indicate that VEGFR-1 is more expressed in high-grade gliomas than in low-grade gliomas [144]. Consistently, glioblastoma specimens and primary cultures of tumor stem cells were found to express VEGFR-1 [145,146]. Jiang et al. demonstrated that VEGFR-1 overexpression increases in vitro and in vivo glioma growth via modulation of the Sonic Hedgehog Homolog (SHH) signaling pathway [147]. VEGFR-1 activation also influences glioblastoma cell migration and ECM invasion. In particular, in U87, LN18, and A172 human glioblastoma cell lines, VEGFR-1 stimulation by both PlGF and VEGF-A resulted in VEGFR-1 phosphorylation at Tyr 1213, followed by downstream phosphorylation of ERK1/2, increased chemotaxis and invasiveness [145]. In vitro treatment with the anti-VEGFR-1 D16F7 mAb markedly inhibited receptor autophosphorylation and ERK1/2 activation and reduced glioblastoma cell invasive behavior [145]. In vivo studies in glioblastoma murine models indicated that D16F7 was well-tolerated and confirmed its promising therapeutic potential, as the mAb induced a decrease in glioma growth and angiogenesis, as well as an increase in mice survival. In particular, the efficacy of D16F7 mAb was tested in heterotopic (intramuscular) and orthotopic (intracranial) models using rat C6 glioma sublines, transfected to overexpress VEGFR-1. In the heterotopic intramuscular model, treatment with D16F7 reduced tumor growth and in the orthotopic intracranial model the mAb increased animal survival by 40% and 65% at 10 and 20 mg/kg, respectively, with a remarkable percentage (46%) of long-term survivors at the higher dose. Additionally, immunohistochemical analysis of tumor sections from D16F7-treated animals showed a higher number of apoptotic cells and fewer blood vessels compared to untreated mice [148].

Of interest, glioblastoma was reported to express higher sVEGFR-1 and VEGF-A levels, compared to low-grade gliomas, and a low sVEGFR-1/VEGF-A ratio has been correlated with higher tumor aggressiveness [31]. Although VEGF-A is important in the switch from low-grade glioma to highly vascularized glioma, the upregulation of sVEGFR-1 in glioblastoma seems a contradiction, given its inhibitory role in angiogenesis. However, the sVEGFR-1/VEGF-A ratio detected in glioblastoma was 2.6-fold lower than in diffuse astrocytoma and thus likely sufficient to induce a shift towards angiogenesis [31].

Glioblastoma-associated microglia/macrophages (GAMs) represent the largest amount of tumor-infiltrating cells and are known to exert protumoral and proangiogenic effects and contribute to bevacizumab resistance [149]. Actually, high VEGF-A levels were reported to down-modulate VEGFR-2 in GAMs and to reduce the infiltration of microglia/macrophages in the tumor mass by approximately 50% [150]. In patients who experienced tumor progression during bevacizumab therapy, an increase of GAMs was observed, which correlated with poor survival [151,152,153]. Therefore, bevacizumab should be combined with therapies targeting GAMs to prevent their expansion. In this regard, by analyzing surgical specimens collected from glioblastoma patients, we recently demonstrated that GAMs expressed VEGFR-1 and the percentage of VEGFR-1 positive GAMs was higher in the tumor tissue than in the surrounding parenchyma [154]. Thus, VEGFR-1 targeting might synergize with anti-VEGF-A therapies counteracting the rebound-proangiogenic effects mediated by GAMs and delaying resistance development.

### 2.5. Melanoma

Melanoma is the most severe type of skin cancer whose incidence is increasing worldwide. Although at an early stage melanoma can be cured by surgical resection, the prognosis of the advanced/metastatic disease is extremely poor [155]. The most common sites of distant metastases are the skin, lung, brain, liver, bone, and intestine. Brain metastases are diagnosed in up to 50% of patients and are associated with a dismal outcome [156].

Melanoma cells have been found to expresses VEGFR-1 together with its ligands PlGF and VEGF-A, and the resulting signaling is able to enhance tumor cell proliferation, chemotaxis, and ECM invasion [157,158]. In particular, VEGF-A and PlGF release was more frequently detected in cell lines derived from metastatic melanomas than in those derived from primary tumors. Besides VEGFR-1, also VEGFR-2, NRP-1, and NRP-2 were expressed in the majority of the melanoma cell lines tested [157]. Cell lines secreting high VEGF-A levels and expressing VEGFR-1 and/or VEGFR-2 showed a spontaneous ECM invasion and inhibition of VEGFR-TK activity abrogated this property. Interestingly, a difference was observed in ECM invasion between two melanoma cell clones which differed in the expression of VEGFR-2, while having comparable levels of VEGFR-1: cells with higher expression of VEGFR-2 were 8-fold more invasive, suggesting that VEGFR-2 plays a relevant role in ECM invasion triggered by VEGF-A in melanoma cells [159].

Most melanoma cell lines derived from primary tumors or lymph node metastases presented higher levels of *sVEGFR-1 mRNA*, in comparison to melanocytes or cell lines generated from cutaneous metastases. The *sVEGFR-1⁄VEGFR-1* transcripts ratio resulted decreased in cutaneous metastases compared to primary melanomas, due to the reduced sVEGFR-1 expression. Therefore, it has been suggested that sVEGFR-1 plays a dual role in the initial phases of melanoma progression: as a soluble isoform regulating tumor cell invasiveness and as an ECM component, directly stimulating the mobilization of endothelial and tumor cells [36].

Treatment of human (CR-Mel) and murine (B16F10) melanoma cells with the anti-VEGFR-1 D16F7 mAb strongly down-modulated the migration triggered by PlGF. Moreover, D16F7 inhibited vasculogenic mimicry (i.e., the formation of tube-like structures, resembling blood vessels) by melanoma cells in response to VEGF-A. In vivo studies performed in a syngeneic murine melanoma model (B16F10 cells injected in B6D2F1 mice) confirmed the efficacy of VEGFR-1 blockade by D16F7 and the good tolerability of the treatment. After 16 days of treatment, mice showed a tumor volume inhibition of 48.6% and 74.4% with 10 mg/kg and 20 mg/kg, respectively. Furthermore, immunohistochemical analysis of melanoma sections from D16F7-treated animals showed a reduction of tumor infiltration by monocytes/macrophages and a marked decrease of bone invasion by melanoma cells [160].

The upregulation of VEGFR-1 in melanoma also contributes to the development of resistance to BRAF inhibitors (BRAFi). In fact, the expression of VEGFR-1 in melanoma cells resistant to the BRAFi vemurafenib was higher than in their BRAFi-sensitive counterparts, whereas the transient silencing of VEGFR-1 in resistant cells increased BRAFi sensitivity and in susceptible cells delayed resistance development. Furthermore, vemurafenib-resistant melanoma cells expressing VEGFR-1 showed a higher invasive behavior, compared to melanoma cells susceptible to the BRAFi. Accordingly, treatment with D16F7 markedly reduced ECM invasion by resistant cells in response to VEGF-A and PlGF, suggesting that VEGFR-1 blockade in combination with the BRAFi might delay the acquisition of a resistance phenotype [161].

Overexpression of PlGF in the B16-BL6 melanoma cell line intradermally injected in the skin of transgenic mice stimulated tumor growth, vascularization, and metastatic spreading [162]. Moreover, PlGF secretion by melanoma cells favored resistance to temozolomide through a mechanism involving NF-κB. Indeed, PlGF silencing or inhibition of NF-κB restored melanoma cell sensitivity to the chemotherapeutic agent [163] Finally, *VEGF-B* transcript levels in the tumor measured in a large cohort of melanoma patients inversely correlated with survival [69].

### 2.6. Bone Cancer

Primary bone cancers are rare (0.2% of all cancer cases) and include osteosarcoma, chondrosarcoma, and Ewing’s sarcoma [164]. Osteosarcoma is the most common primary bone tumor in childhood and adolescence. Although it is a rare tumor, it accounts for ~6% of all cancer cases among patients with less than 20 years of age and is the third most common malignancy in adolescence. In adults, it represents < 1% of all newly diagnosed cancer types. Conversely, chondrosarcoma mainly affects patients aged 40 to 70 years. Ewing sarcoma is the second most common tumor in children and adolescents. Approximately 15–20% of osteosarcomas are metastatic at the time of diagnosis and the metastatic or recurrent disease is associated with a very poor prognosis. The most common metastatic sites are the lungs (~90% of all cases), other bones, and lymph nodes [165,166]. In the case of chondrosarcoma, skeletal and lung metastases are detected at diagnosis or within 12–18 months in the case of dedifferentiated chondrosarcoma (10% of cases), whereas Ewing sarcoma has a high propensity to metastasize to the lungs, bone and bone marrow [167,168,169].

In osteosarcoma patients, VEGF-A serum levels are increased and associate with enhanced vessel density in the tumor and a decreased survival [170,171,172]. Moreover, a meta-analysis study performed in a Chinese population reported a link between *VEGF-A* gene polymorphisms (VEGF +936C/T and –634 G/C) and the risk of developing osteosarcoma [173].

In hypoxic conditions, VEGF-A expression is promoted by HIF-1α in U2-OS osteosarcoma cells, and VEGF-A is able to stimulate tumor cell invasiveness. Indeed, HIF-1α and VEGF-A knockdown decreased the invasive potential of Saos-2 and U2-OS osteosarcoma cell lines [174].

In regard to the involvement of VEGFR-1 in the VEGF-A-mediated effects on osteosarcoma malignant behavior, a constitutive activation of an autocrine VEGF-A/VEGFR-1 signaling pathway was reported in highly aggressive osteosarcoma [175]. The malignant potential was measured in terms of growth rate and formation of spontaneous lung metastases in in vivo murine models. These findings were obtained comparing aggressive murine and human osteosarcoma cell lines (K7M3 and 143B, respectively) with their parental cell lines (K12 and TE85, respectively). In particular, the expression of both VEGF-A and VEGFR-1 was more abundant in K7M3 and 143B cells than in K12 and TE85 cells. Conversely, VEGFR-2 expression was not detected in these osteosarcoma cell lines. To assess the effect of VEGFR-1 expression on osteosarcoma growth in vivo, separated K7M3 subpopulations, expressing high or low receptor levels, were injected into the mouse tibias. High VEGFR-1 expressing K7M3 cells originated significantly larger tumors than low VEGFR-1 expressing cells. Consistently with the in vitro data, tumors originated from high-VEGFR-1 K7M3 cells produced more VEGF-A than low-VEGFR-1 cells [175].

The miR-134, whose expression is down-modulated in osteosarcoma tissues [176], was found to attenuate the growth and neovessel formation in osteosarcoma by targeting the VEGF-A/VEGFR-1 signaling [177]. In human osteosarcoma, MG-63, U2-OS, and Saos-2 cells miR-134 levels were significantly lower than in osteoblasts; on the other hand, miR-134 overexpression inhibited the proliferation of tumor cells and significantly increased apoptosis. In addition, human umbilical vein endothelial cells (HUVECs) cultured in conditioned medium from miR-134 overexpressing Saos-2 cells showed a significant inhibition of tube formation potential. When injected in BALB/c nude mice, miR-134 stably expressing Saos-2 cells formed smaller and less vascularized tumors. Western blot analysis indicated that VEGF-A and VEGFR-1 expression in the Saos-2/miR-134 tumors were lower than those in the Saos-2/control tumors and a bioinformatic analysis validated these in vitro and in vivo data, by indicating that the 3′-UTR regions of *VEGF-A* and *VEGFR-1* transcripts contain the motif for miR-134 binding [177].

MiR-1 is another miRNA significantly down-modulated in osteosarcoma tissues as well as in Saos-2 and U2-OS cell lines. U2-OS cells overexpressing miR-1 showed a significant suppression of tumor growth and migration/invasion potential, in comparison to control cells. Also in this case, a luciferase assay demonstrated that miR-1 directly inhibited VEGF-A expression at the post-transcriptional level, by binding to its mRNA 3′-UTR region [178].

High VEGF-A levels, and, in particular, the VEGF-A165 isoform, were also reported in Ewing sarcoma and appeared to contribute to stimulate the osteolytic process [179,180]. The mechanism underlying this VEGF-A effect, consisted in the upregulation of the receptor activator of nuclear factor kappa-Β ligand (RANKL), whose interaction with RANK represents a well-known signaling pathway stimulating osteoclastogenesis and bone resorption. Moreover, VEGF-A increased the recruitment in the tumor of TAMs, which are capable of differentiating into osteoclasts and contribute to inflammation and angiogenesis [181]. In fact, TAM infiltration is further enhanced by other cytokines released by TAMs themselves and stimulate the release of VEGF-A by tumor cells. Consistently, in human Ewing sarcoma samples, TAM infiltration was associated with enhanced tumor vascularization and poor OS [181].

In chondrosarcoma, VEGF-A expression was found to correlate with adiponectin expression and tumor stage [182]. In this tumor type, the high expression of WNT1-inducible signaling pathway protein-3 (WISP-3) stimulated angiogenesis by downregulating miR-452 that in turn is able to inhibit the expression of VEGF-A [183].

### 2.7. Pancreatic Cancer

Pancreatic cancer is the fourth leading cause of cancer death, with 1-year and 5-year survival rates of 25% and 5%, respectively. Ninety-five percent of cases occur within the exocrine portion of pancreas and ductal adenocarcinoma accounts for ∼80% of all pancreatic cancers [184]. Approximately 50% of patients are diagnosed with metastatic disease, and the liver is the most common metastatic site followed by the lung and peritoneum, although metastases to the bone, adrenal gland, and distant lymph nodes have also been detected [185,186].

VEGF-A is endowed with a primary role in human pancreatic cancer angiogenesis and metastasis [187]. By immunohistochemistry and in situ hybridization analysis VEGF-A was found to be expressed in ductal epithelial tumor cells, but not in ductal cells of non-transformed pancreas or chronic pancreatitis [187,188]. VEGF-A role in pancreatic cancer progression also influences tumor cells glucose metabolism: it enhances glycolysis via HIF-1α upregulation and NRP-1 co-receptor involvement. Indeed, pancreatic cancer cells stimulated with VEGF-A165 showed a metabolic transition from mitochondrial oxidative phosphorylation to glycolysis. HIF-1α and NRP-1 protein levels were both increased after VEGF-A165 stimulation and *NRP-1* silencing by shRNA reduced glycolysis in pancreatic cancer cells [189]. Increased levels of PlGF were reported in obesity-associated pancreatic cancer patients and ablation of the VEGFR-1 signaling in pancreatic ductal adenocarcinoma murine models prevented obesity-induced tumor progression [77].

Immunostaining with antibodies directed against VEGFR-1 and VEGFR-2 showed expression of these receptors in 29% and 43% of pancreatic carcinoma tissues, respectively. VEGF-A mRNA and protein were also detected by RT-PCR and ELISA, respectively, in AsPc-1, Capan-1, Capan-2, Dan-G, and Panc-1 human ductal pancreatic carcinoma cell lines, whereas VEGFR-1 expression was observed only in three of these cell lines (AsPc-1, Dan-G, and Panc-1) [188]. In another study, VEGFR-1 and VEGFR-2 were detected in 90% and 65% of cancer cases, respectively, and their coexpression was recognized as a poor prognostic factor [190]. Furthermore, the analysis of tissue samples from another cohort of pancreatic patients revealed that VEGFR-1 and VEGFR-3 expression was significantly higher in tumor cells and tumor-associated endothelial cells, while VEGFR-2 was detected only in tumor cells. Of interest, the triple combination of VEGFRs artificial miRNAs, able to concurrently inactivate the three VEGFR isoforms, in pancreatic cancer cell lines (BXPC-3, MIAPACA2, Panc-1, and SW1990) and in mouse pancreatic cancer xenograft models (SW1990 cells), reduced proliferation, migration and invasion, and increased apoptosis. Moreover, triple VEGFRs downregulation synergized with standard chemotherapy (5-fluorouracil and cisplatin combination) in vivo [191].

Table 1 summarizes data described in Section 2.1, Section 2.2, Section 2.3, Section 2.4, Section 2.5, Section 2.6 and Section 2.7.

### 2.8. Cancers of the Gastrointestinal Tract

#### 2.8.1. Oral Cancer

Oral squamous cell carcinoma (OSCC) (~90% of all oral tumors) mainly affects the buccal mucosa or the tongue (32% and 22% of total cases, respectively), and only in 3% of total reported cases, the gingiva. Distant metastases strongly influence the prognosis of OSCC and the most common primary sites are hypopharynx, followed by the tongue and lymph nodes [192,193].

A meta-analysis study has shown that VEGF-A overexpression in patients with OSCC, adenoid cystic carcinoma, and mucoepidermoid carcinoma of the salivary glands, correlated with a poor prognosis [194]. These data were confirmed by the results of The Cancer Genome Atlas (TCGA) dataset search and by immunohistochemistry analysis in surgical sections from oral cancer patients [195]. Moreover, tumor recurrence and lymph node metastases in gingival cancer positively correlated with an increased expression of VEGF-A [196].

The VEGFR-1 signaling pathway triggered by VEGF-A seems to be important for bone invasion by OSCC cells [197]. Activation of the VEGFR-1 signaling pathway in pre-osteoclasts, after binding of OSCC-derived VEGF-A, would lead to their differentiation and migration to bone-resorbing areas. Alternatively, in OSCC cells the VEGF-A/VEGFR-1 signaling would upregulate RANKL, with consequent stimulation of the osteolytic process [197].

#### 2.8.2. Esophageal Cancer

Esophageal cancer, which comprises esophageal squamous cell carcinoma and adenocarcinoma, is the eighth most common cancer type. Esophageal cancer standard therapy is surgical or endoscopic resection and chemoradiotherapy, but within 5 years about 50% of patients develop disease recurrence, due to hematogenous distribution of circulating tumor cells to distant sites (i.e., liver, lymph nodes, lung, bone, and brain) [198,199,200].

Patients with VEGF-A overexpression have a significantly increased risk (2-fold) of disease progression, with the development of distant metastases and shorter OS [196]. Consistently, a study in patients up to 10 years following radiochemotherapy, showed a trend towards worse survival when tumor cells expressed VEGF-A and VEGFR-1 [201]. Furthermore, a clinical study enrolling 334 patients with advanced esophageal squamous cell carcinoma revealed that the genetic polymorphism rs2010963 in *VEGF-A* gene independently correlated with worse OS, although this genotype was not associated with high pretreatment VEGF-A levels in the serum [202].

#### 2.8.3. Gastric Cancer

Malignant tumors affecting the stomach represent the fifth most common type of cancer and one of the leading causes of cancer-related death. The liver and peritoneum are the most frequent metastatic sites of gastric cancer, followed by the lung and bone [203,204].

VEGFR-1 expression within the bone marrow at tumor-specific pre-metastatic sites and in peripheral blood samples of patients with gastric cancer was found to be important for the formation of hematogenous metastases. Indeed, Mimori et al. reported a high probability of tumor recurrence and development of hematogenous metastases in stages II and III patients, if both high expression of VEGFR-1 and isolated tumor cells were present [205]. A recent meta-analysis has suggested that also VEGFR-2 expression levels are associated with poor prognosis [206].

The evidence of VEGF-A involvement in gastric cancer metastases derives from the demonstration that increased VEGF-A levels in cancer tissues correlate with higher expression of collapsin response mediator protein family 4 (CRMP4), a protein involved in the metastatic process [207]. Moreover, VEGF-A levels resulted significantly high in both serum and plasma of patients with gastric cancer, and decreased after tumor excision, suggesting that the angiogenic factor was mainly secreted by the tumor mass [208].

Regarding the selective VEGFR-1 ligand PlGF, a direct correlation between the expression of this growth factor and tumor cell viability, proliferation, and migration ability has been reported in the human gastric adenocarcinoma AGS cell line [209]. The same study demonstrated, by gene silencing experiments, that PlGF exerts its roles through PI3K/AKT and p38 MAPK signaling pathways [209].

#### 2.8.4. Colorectal Cancer

Colorectal cancer is the third most common cancer, with metastases being the major cause of death. Up to 60% of patients develop distant metastases within 5 years and the common metastatic sites are the liver and the peritoneum, followed by the lung and bone [210,211].

VEGFR-1 was detected in colorectal cancer, and the interaction with its ligands enhanced the migration/invasion ability of tumor cells. In particular, VEGFR-1 activation in response to VEGF-A or VEGF-B binding promoted phenotypic changes associated with tumor progression and metastases [212]. Activation of the ERK-1/2 and JNK MAPK downstream signaling pathways was identified as the mechanism leading to cell migration, invasion, and colony formation. These effects were only mediated by activation of VEGFR-1 and not by other receptors, as they were specifically inhibited by treatment with the anti-VEGFR-1 neutralizing IMC-18F1 mAb [212]. Consistently, Lesslie et al. observed a marked increase in cellular migration and tyrosine phosphorylation of focal adhesion kinase (FAK), paxillin, and p130cas, following stimulation of VEGFR-1 by VEGF-A [213]. Moreover, colorectal cancer cells expressing PlGF and VEGFR-1 had higher invasive/chemotactic ability, due to phosphorylation of p38 MAPK and upregulation of MMP9 expression [214]. The relevant role of VEGFR-1 in colon cancer was further confirmed by the results of a recent study showing that selective blockade of VEGFR-1 by a receptor specific peptide (iVR1) in syngeneic and xenograft colorectal cancer models markedly inhibited tumor growth and recruitment of monocyte/macrophages at the tumor site [215]. The important role of PlGF in colon cancer progression has been also demonstrated in a study on patients who underwent surgery for primary colorectal cancer resection. Immunohistochemistry analysis of resected tumor tissue samples showed that PlGF expression was more commonly observed in the presence of lymph node metastases than in the absence of lymphatic involvement, and correlated with a poor prognosis [216]. Immunohistochemical analysis of cancer tissues revealed VEGF-B expression mostly in intratumoral vessels but also in tumor cells which correlated with hematogenous metastases [217].

An intracrine VEGF-A/VEGFR-1 signaling was found to mediate the survival of colorectal cancer cells, as indicated by the decrease of cell survival (reduced proliferation/increased apoptosis) and the increase of sensitivity to chemotherapy shown by the human colorectal cancer HCT116, SW480, and HT29 cell lines, after *VEGF-A* or *VEGFR-1* silencing through RNA interference [218]. Indeed, in colorectal cancer cells, the targeting of extracellular VEGF-A with high doses of bevacizumab did not affect cell migration, whereas intracellular VEGF-A depletion significantly impaired migration and ECM invasion. The reduced tumor cell viability was attributed to a disrupted AKT and ERK1/2 signaling. On the other hand, inhibition of paracrine or autocrine VEGF-A signaling did not affect the levels of phosphorylated-AKT and -ERK1/2. The intracrine VEGF-A/VEGFR-1 signaling pathway seemed to regulate cell migration, probably through modulation of FAK activity [219].

Noteworthy, a recent study on metastasis-associated macrophages (MAMs) suggested that circulating VEGFR-1 expressing monocytes might act as a biomarker to predict colorectal cancer recurrence in the liver. Indeed, in patients with metastatic colorectal cancer, a correlation was found between macrophage infiltration and vessel density in the hepatic metastasis but not in the primary tumor. Moreover, MAMs expressed an M2-like phenotype (i.e., anti-inflammatory/proangiogenic) and overexpressed VEGFR-1, as demonstrated by qPCR, flow cytometry, and immunofluorescence analysis. Consistently, high VEGFR-1 expressing MAMs induced a marked angiogenic response in an in vitro sprouting assay, compared to low VEGFR-1 expressing MAMs. The implication of VEGFR-1-positive myeloid cells in colon cancer metastatic growth and angiogenesis in the liver was confirmed in an in vivo murine model where *VEGFR-1* was selectively knocked down in hematopoietic stem cells [220].

Table 2 summarizes data described in Section 2.8.

### 2.9. Sex-Specific Cancers

#### 2.9.1. Breast Cancer

Breast cancer is the most frequent malignant tumor in women worldwide, associated with a high frequency of recurrence and metastases, which represent the major cause of mortality. Breast cancer preferentially metastasizes to the bone and lung via the lymphatic system [221,222]. It has been reported that breast cancer cells are able to modify the microenvironment of the lung in a pre-metastatic phase, inducing tight junction disruptions and pulmonary vascular hyperpermeability, thus efficiently promoting extravasation, which is the first key step in metastasis formation [223,224]. The VEGF-A-protein kinase C (PKC) pathway seemed to be required for this pre-metastatic destabilization of pulmonary tight junctions [225]. Approximately 15% of breast cancer cases develop liver metastases [226]. A study based on bioinformatics and microarray gene expression analysis, comparing data from liver aggressive specimens and primary tumor specimens, revealed the differential expression of 48 genes. In particular, VEGF-A as well as the MAPK and NF-κB signaling pathways were identified as the key players in breast cancer liver metastases [227].

Among oncogenic miRNAs regulating tumor growth and metastasis formation, MiR-126 reduced cell proliferation and VEGF-A levels when overexpressed in the human breast cancer MCF-7 cell line [228]. On the other hand, overexpression in MCF-7 cells of COX-2-induced miRNAs, miR526b and miR655, resulted in the upregulation of VEGF-A, VEGF-C, and VEGF-D, and of their VEGFR-1 and VEGFR-2 receptors. Furthermore, exposure of HUVECs to conditioned media from these miRNAs overexpressing cells stimulated chemotaxis and capillary-like formation. Accordingly, an in situ analysis of tumor specimens confirmed the correlation between miR526b/miR655 expression and significantly higher levels of VEGF-A and VEGF-D compared to non-adjacent control tissues [229].

VEGF-A signaling pathway has been also involved in hydrogen sulfide (H_2_S)-induced angiogenesis [230]. Cystathionine-γ-lyase (CSE), a key enzyme involved in the endogenous H_2_S production [231], was found upregulated in samples from breast cancer patients with lymph node metastases and in metastatic breast cancer cell lines. In breast cancer metastases, CSE behaved as a positive regulator of VEGF-A expression and induced the PI3K/AKT, FAK-paxillin, and RAS-MAPK-ERK1/2 pathways. Overexpression of CSE also increased the levels of MMP2 and MMP9 in early metastatic breast cancer cells, allowing the degradation of the ECM and consequently the entering of tumor cells into the blood circulation [232].

PlGF is expressed by 30–50% of primary human breast cancers [233] and stimulates tumor cell motility in vitro, through activation of intracellular signaling cascades, including ERK1/2 function and cytoskeletal remodeling. As expected, the PlGF-mediated chemotaxis was abrogated by a VEGFR-1-antagonist peptide, BP-1, and by an anti-PlGF antibody [233,234]. PlGF from breast cancer cell lines also appeared as a critical factor in the promotion of CD34^+^ hematopoietic progenitor differentiation into tumor-mobilized CD11b^+^ myeloid cells, endowed with proangiogenic effects (i.e., formation of capillary-like tube structures in vitro and neovessels in vivo). In particular, human CD34^+^ progenitors, cultured in conditioned medium from the human metastatic breast MDA-MB-231 cancer cell line, generated CD11b^+^ cells that were able to induce HUVECs sprouting in vitro. This effect was not observed when CD34^+^ progenitors were exposed to conditioned medium collected from the human normal breast epithelium-derived MCF10A cell line. The addition of the anti–VEGFR-1 KM1732 mAb and a VEGFR-1 trap (sFlt-1-Fc) to CD34^+^ cultures, pretreated with the MDA-MB-231-conditioned medium or PlGF, prevented the generation of CD11b^+^ cells with sprouting-inducing ability [235]. Furthermore, CD11b^+^ cells induced a significant angiogenic response in the murine corneal angiogenesis in vivo assay and PlGF silencing reduced the proangiogenic activity of circulating CD11b^+^ myelomonocytic cells in a breast cancer murine model. Consistently with these results, high levels of PlGF and proangiogenic CD11b^+^ myelomonocytes were detected in the peripheral blood of breast cancer patients, but not in samples from healthy controls [235].

Obesity has been associated with unfavorable survival in breast cancer patients [236] and the PlGF/VEGFR-1 signaling has been also involved in obesity-induced breast cancer progression [77]. In particular, in diet-induced obese VEGFR-1 TK-null mice orthotopically implanted with syngeneic E0771 breast cancer cells, a decrease in tumor progression and lung metastases was observed, compared to lean animals. Furthermore, while obesity promoted IL-6 and MMP9 expression in tumors, deletion of VEGFR-1 TK domain decreased the expression of these pro-M2 markers only in obese mice. PlGF was identified as the VEGFR-1 ligand responsible for such effects since its plasma levels were elevated in diet-induced obese mice, and its deletion induced similar effects to those observed in VEGFR-1-TK-null obese mice. Consistently with these data obtained in the murine model, the plasma levels of PlGF were found to correlate with the body-mass-index in obesity-associated breast cancer patients [77].

#### 2.9.2. Ovarian Cancer

Ovarian cancer represents ~3% of all cancers affecting women, but it is often more aggressive than other malignancies of the female reproductive tract [237,238]. Tumor spreading mainly occurs by the intraperitoneal, lymphatic, and hematogenous routes and the most common metastatic sites are peritoneum, lymph nodes, and liver, and sometimes the bone and brain. Compared with benign ovarian tumor tissues, malignant cancer tissues show a significantly higher expression of VEGF-A, which is considered as an unfavorable prognostic factor [239]. Genotoxic agents like mitomycin C have been found to induce an antiangiogenic splice isoform of VEGF-A, i.e., VEGF111b, in human ovarian cancer cell lines, thus inhibiting angiogenesis [240] and tumor growth, both in vitro and in vivo [241]. Also, VEGF-A expression in ovarian cancer cells has been considered a poor prognostic factor in light of its influence on tumor immune evasion, via the recruitment and activation of myeloid-derived suppressor cells [242]. Ovarian cancer specimens also showed increased levels of both PlGF and MMP7, compared to normal ovarian tissues. The MMP7 increase was attributed to the PlGF-induced downregulation of miR-543, a miRNA that interacts with the 3′-UTR region of *MMP7* mRNA and inhibits its translation [243]. Also ZEB2 expression, a transcription factor with a crucial role in EMT [244], strongly correlated with PlGF levels in ovarian cancer tissues: PlGF overexpression significantly increased ZEB2 levels and cell invasiveness, conversely PlGF depletion was associated with a decline of ZEB2 levels and cell invasiveness. This effect resulted to be p38 MAPK-dependent, since it was abolished by a specific p38 MAPK inhibitor, SB203580 [245].

Of interest, ovarian cancer cells transfected with a plasmid encoding sVEGFR-1 (pLV-sFLT-1) showed the typical features of necrotic cells, i.e., cell swelling, plasma membrane rupture, and release of cell content. Mice transplanted with human ovarian adenocarcinoma SKOV3 cells transfected with pLV-sFLT1 or exogenously (intraperitoneally) treated with recombinant sVEGFR-1, confirmed the antitumor effect of sVEGFR-1 also in vivo, in terms of reduced tumor size compared to control animals. Moreover, none of these approaches induced significant adverse effects [246].

#### 2.9.3. Cervical Cancer

Cervical cancer is the fourth cancer in women with a significant metastatic spreading, involving hematogenous as well as lymphatic dissemination. The lung, followed by the bone, liver, and brain are the main target organs of hematogenous spreading [247].

VEGFR-1 as well VEGFR-2 expression levels in biopsy specimens have been recognized as prognostic factors for patients with cervical cancer: high VEGFR-1 expression was linked to distant metastases, together with poor OS and PFS, whereas high VEGFR-2 expression correlated with increased tumor size and reduced OS [248].

VEGF-A and VEGF-B expression in the cytoplasm of cervical cancer cells was not associated with the expression of their receptors and patients’ survival [248]. On the other hand, a recent study reported that patients with high serum levels of both VEGF-A and VEGFR-2 presented bulky tumors, pelvic lymph node involvement, parametrial infiltration, and significantly lower OS than patients with low VEGF-A and VEGFR-2 expression [249]. High expression of VEGF-A and VEGFR-1 was also identified in tumor specimens obtained from patients with post-radiotherapy relapsed/persistent cervical cancer. These data suggest that subpopulations of cervical cancer cells with higher VEGF-A levels survive, and once selected by chemoradiation, give rise to tumor recurrence. Moreover, ionizing radiations can themselves induce HIF-1α and, consequently, VEGF-A expression [250].

Deregulation of cellular energetic metabolism has been recognized as another feature of cancer, aimed at increasing the production of lactate, whose efflux in the ECM induces angiogenesis. In a recent analysis of 232 cervical adenocarcinoma samples, VEGF-A was reported to be coexpressed with metabolism-related proteins (monocarboxylate transporters (MCT)), in particular, with the MCT4 isoform, allowing the conclusion that the angiogenic switch and the metabolic remodeling synergistically act to affect cervical adenocarcinomas aggressiveness [251].

Similarly to what observed in other cancers, PlGF was shown to induce the molecular modifications of EMT also in cervical cancer, as demonstrated in the human SiHa cell line, where the growth factor promoted migration and metastases through activation of the ERK/MAPK signaling pathway [252]. Moreover, cervical cancer specimens typically presented a dense infiltrate of TAMs, which in turn was associated with a poor prognosis [253]. Culture supernatants obtained from cervical cancer cell lines (HeLa, SiHa, and C-33A) were found to contain VEGF-A, and when its effect was assessed on THP-1 macrophages, these cells developed the M2-like phenotype. Furthermore, M2 macrophages continued to produce VEGF-A 48 h after removal of the supernatant, confirming their role in controlling cervical cancer growth, angiogenesis and metastasis [254].

#### 2.9.4. Prostate Cancer

Prostate cancer is the second most common malignancy occurring in men, after lung cancer. Radical prostatectomy and radiotherapy represent the currently recommended treatments for localized tumors, but up to 30% of patients experience local recurrence or develop a still incurable metastatic disease [255]. Bone metastases are the main cause of mortality in patients with prostate cancer in an advanced stage and an acidic extracellular microenvironment has been shown to favor their development. In fact, PC-3 prostate cancer cells, grown in an acidic medium (pH 6.5), showed increased viability, spheroid formation, and expression of stem cell-related markers, compared to cells grown in a neutral medium (pH 7.4). Moreover, the acidic medium stimulated VEGF-A and MMP9 secretion and promoted cell invasiveness. The same acidic conditioned medium obtained from PC-3 cell culture triggered the ability of bone marrow-derived endothelial progenitor cells to generate blood vessels, by increasing their survival, migration, and tube formation potential. The underlying molecular mechanism was identified in the autophosphorylation of VEGFR-2, followed by the phosphorylation of its downstream AKT and p38 target proteins [256]. Treatment with cabozantinib, a multi-targeted kinase inhibitor of VEGFR-1, VEGFR-2, and VEGFR-3 and several other kinases (i.e., AXL, MET, RET, KIT, FLT3, ROS1, MER, TYRO3, TRKB, and TIE-2), was shown to reduce prostate cancer growth in the bone, by suppressing the osteoblastic activity, a prerequisite for bone invasion, in an in vivo preclinical model. Mice injected in tibiae with cabozantinib-resistant prostate cancer PC-3 and C4–2b cells showed a decrease of tumor-associated osteoclasts and a reduction of osteolysis, after 3 weeks of treatment with cabozantinib. As the culture of cancer cells isolated from tumor-bearing mice showed no changes in IC_50_ values after the in vivo growth, cabozantinib effects were attributed to alterations in the tumor microenvironment [257].

Another study demonstrated that the promotion of angiogenesis and prostate cancer metastases may occur not only through the raise of VEGF-A levels but also through the reduction of class 3 semaphorins. In particular, although VEGF-A expression was increased only in metastatic tumors, class 3 semaphorin expression was formerly reduced in primary tumors, thus anticipating a favorable condition for the subsequent metastatic phase [258]. Consistently, immunohistochemical analysis of patient specimens showed that prostate cancer samples express higher levels of VEGF-A, VEGFR-1, and VEGFR-2 than benign prostatic hyperplasia (BPH) and high-grade prostate intraepithelial neoplasia (HGPIN) samples. Microvascular density was also higher in prostate cancer, compared to BPH and HGPIN, whereas no differences were detected between BPH and HGPIN. In detail, when the analysis was restricted to prostate cancer samples, VEGF-A expression and microvascular density were higher in poorly differentiated tumors and in the presence of perineural invasion [259].

Concerning the potential regulatory role exerted by miRNAs in tumor invasion and metastases, human prostate cancer specimens and cell lines showed a significant downregulation of miR-130b [260]. When ectopically expressed, miR-130b blocked prostate cancer angiogenesis, both in vitro and in vivo, and stimulated the proliferation, invasion and capillary-like formation of HUVECs, by attenuating NF-κB signaling and *VEGF-A* transcription. Consistently, ectopic VEGF-A expression decreased miR-130b level and abrogated its antiangiogenic effect, thus promoting the angiogenic response [261].

In addition to lowered oxygen, elevated androgens have also been found to play a crucial role in upregulating VEGF-A levels [262]. Mechanistically, it seems that the activated androgen receptor binds to Sp1 (a zinc-finger transcription factor that specifically interacts with GC-rich promoter regions) and the resulting complex then binds to the core promoter region of the *VEGF-A* gene, inducing its transcription [263].

VEGF-A has also been found to inhibit the maturation and activity of DCs, and this inhibition consistently increased with tumor grade. In particular, the infiltration of VEGF-A positive cells inversely correlated with the maturation and activity of DCs. In fact, both S100, a protein present in all DCs, and CD208, expressed by mature and functional DCs, were found to be lower in prostate cancer than in BPH and prostatic intraepithelial neoplasia [264].

Table 3 summarizes data described in Section 2.9.

### 2.10. Leukemia

In addition to solid tumors, VEGF-A and PlGF play a relevant role in angiogenesis occurring within the bone marrow microenvironment and in acute leukemia spreading at the central nervous system (CNS) site [265,266,267,268,269]. Acute myeloid leukemia (AML) is the most common acute form diagnosed in adults and is characterized by an increased number of immature myeloid cells in the bone marrow, whereas acute lymphocytic leukemia (ALL) is a malignancy of immature lymphocytes that represents 60–74% of all leukemia cases in patients under the age of 20 [270].

High VEGF-A levels in the cerebrospinal fluid of leukemia patients have been implicated in CNS metastasis [271]. In ALL xenograft and syngeneic mouse leukemia in vivo models, VEGF-A produced by leukemia cells was required for their trans-endothelial migration and penetration into the CNS [272,273].

Conflicting data have been reported regarding the VEGFR-1 and VEGFR-2 expression in these two subtypes of leukemia. According to previous studies, the VEGFR-1 was found to be preferentially expressed in bone marrow samples and cell lines from ALL, whereas the VEGFR-2 was expressed mainly in AML cells [274,275,276]. Nevertheless, in myeloid cell lines (i.e., HL-60 and HEL) and in primary leukemia samples of non-specified origin both VEGFR-1 and VEGFR-2 were reported and were functionally activated upon cell exposure to VEGF-A. Moreover, VEGFR-2 was involved both in cell proliferation and migration, whereas VEGFR-1 only in cell migration, as demonstrated by in vitro blockade of VEGFR-2 by the IMC-1C11 mAb and of VEGFR-1 by the clone 6.12 mAb [277]. In agreement with these findings, PlGF or VEGF-A activation of VEGFR-1 in AML HL-60 and HEL cells was implicated in cell migration and only marginally in cell proliferation; furthermore, PlGF was more potent than VEGF-A in promoting this effect [278]. Selective VEGFR-1 stimulation by PlGF of AML cell lines and AML bone marrow samples was associated with the formation of membrane protrusions, induction of p38 and ERK1/2 phosphorylation, activation of the actin-binding protein cofilin, and formation of caveolae-like structures [278]. On the other hand, a different study in myeloid HL60 and KG cells showed that activation of VEGFR-1 by PlGF or by an agonist Ab was able to induce tumor cell proliferation [279]. Treatment with an anti-VEGFR-2 mAb of immunodeficient mice transplanted with HL-60 cells inhibited leukemia growth; however, the in vivo activity of the anti-VEGFR-1 mAb was not tested [277]. Other studies have shown that plasma VEGF-A levels and VEGFR-1 and VEGFR-2 proteins were higher in AML than in ALL and that VEGFR-1, although expressed also in ALL, was the receptor subtype most abundantly expressed in chronic and acute leukemia of myeloid origin [280,281]. Of note, VEGF-A and VEGFRs expression were markedly increased in relapsed/refractory patients compared to remission patients or healthy volunteers. PlGF plasma levels were also higher in samples from patients with blast crisis of chronic myeloid leukemia (CML) compared to the chronic phase. Moreover, in in vivo murine models, PlGF produced by bone marrow stromal cells stimulated angiogenesis in the bone marrow as well as CML cell growth, and treatment with the anti-murine PlGF mAb 5D11D4 prolonged the survival of CML bearing mice [282].

In ALL, VEGFR-1 activation in vitro had scarce effect in cell proliferation but mediated migration and, in an in vivo model, favored the exit of leukemia cells into the bloodstream and the infiltration of extra-medullary sites such as the spleen or other organs [281]. Thus, the authors suggested that blockade of VEGFR-1 by a selective therapeutic agent might counteract leukemia cell movement within the bone marrow, delaying the extra-medullary tumor growth [281]. Furthermore, PlGF stimulated the growth of Philadelphia chromosome positive ALL cells by both autocrine and paracrine pathways [283].

## 3. Currently Approved Antiangiogenic Therapies and Experimental Agents Targeting VEGFR-1 or PlGF

The clinically approved antiangiogenic agents used for the treatment of different types of recurrent, unresectable, or advanced/metastatic solid tumors include:

(i) the humanized mAb bevacizumab, which targets all isoforms of VEGF-A, thereby preventing the activation of both VEGFR-1 and VEGFR-2. This mAb is used in combination with chemotherapy for unresectable/locally advanced, recurrent, or metastatic NSCLC, metastatic colorectal cancer, persistent, recurrent, or metastatic cervical cancer; recurrent ovarian, fallopian tube, and peritoneal cancer; metastatic breast cancer (the latter indication is approved only by EMA), or with interferon alfa for metastatic RCC; and as monotherapy for recurrent glioblastoma (only FDA-approved);

(ii) the fully human mAb ramucirumab, which recognizes the VEGFR-2 preventing its interaction with VEGF-A. Ramucirumab is used in combination with chemotherapy for advanced gastric or gastro-esophageal junction adenocarcinoma, metastatic colorectal cancer, metastatic NSCLC, and as a single agent for advanced or unresectable hepatocellular carcinoma;

(iii) the chimeric protein ziv-aflibercept, in which the second Ig-like domain of VEGFR-1 and the third Ig-like domain of VEGFR-2 are fused to the Fc portion of a human IgG1. Ziv-aflibercept acts by sequestering PlGF, VEGF-B, and all isoforms of VEGF-A; it is approved for metastatic colorectal cancer combined with irinotecan, 5-fluorouracil, and folinic acid;

(iv) small molecules acting as RTK inhibitors, which interact with the catalytic domain of VEGFRs and other growth factor TK receptors. Approved small-molecule kinase inhibitors targeting VEGFRs include axitinib (advanced RCC); cabozantinib (advanced RCC and hepatocellular carcinoma previously treated with sorafenib); erdafitinib (only FDA-approved for metastatic urothelial carcinoma); lenvatinib (locally recurrent or metastatic, progressive, radioactive iodine-refractory differentiated thyroid cancer, advanced RCC, unresectable hepatocellular carcinoma and advanced endometrial carcinoma (the latter indication is only FDA-approved)); pazopanib (renal cell carcinoma and soft tissue sarcoma); regorafenib (metastatic colorectal cancer, unresectable or metastatic gastrointestinal stromal tumors, and hepatocellular carcinoma previously treated with sorafenib); sorafenib (unresectable hepatocellular carcinoma, advanced RCC and progressive, locally advanced or metastatic differentiated thyroid carcinoma refractory to radioactive iodine treatment); sunitinib (unresectable and/or metastatic malignant gastrointestinal stromal tumor after disease progression or intolerance to imatinib mesylate, advanced RCC, progressive, unresectable, or metastatic well-differentiated pancreatic neuroendocrine tumors); tivozanib (advanced RCC); and vandetanib (unresectable locally advanced or metastatic medullary thyroid cancer).

Other approved agents that are endowed with antiangiogenic effects but do not directly target the VEGFRs or their ligands include (i) the mammalian target of rapamycin (mTOR) inhibitors temsirolimus (approved for RCC and mantle cell lymphoma) and everolimus (approved for advanced kidney and breast cancers, subependymal giant cell astrocytoma, pancreatic neuroendocrine tumors, neuroendocrine tumors of gastrointestinal or lung origin), and the (ii) the immunomodulatory drugs (IMiDs) thalidomide, lenalidomide and pomalidomide, approved for the treatment of multiple myeloma (lenalidomide also for relapsed or refractory mantle cell lymphoma and myelodysplastic syndromes with deletion of the long arm of chromosome 5). The mTOR inhibitors and IMiDs mainly act through inhibition of the secretion of VEGF-A and other angiogenic factors such as basic fibroblast growth factor (FGF) or the platelet derived growth factor-A (PDGF-A) [284,285,286].

Overall, the antiangiogenic agents approved so far for cancer treatment inhibit the VEGF-A signaling through both VEGFR-2 and VEGFR-1 (i.e., bevacizumab, ziv-aflibercept, and multi-targeted TK inhibitors), or solely through VEGFR-2 (i.e., ramucirumab). The antibodies targeting exclusively VEGFR-1 are still in a preclinical stage of development, and only one of them has reached Phase I/II clinical trials, so far. Most of these mAbs prevent the interaction of VEGFR-1 ligands with the receptor (competitive inhibitors), whereas only D16F7 blocks receptor signal transduction without inhibiting ligand binding (non-competitive inhibitor) [67]. In particular, anti-human VEGFR-1 mAbs include the following.

(i) The fully human IMC-18F1/icrucumab neutralizing mAb, which blocks the interaction of VEGFR-1 with VEGF-A, VEGF-B, and PlGF. This mAb has shown antitumor activity in human breast carcinoma xenograft models (i.e., DU4475, MDA-MB-231, and MDA-MB-435). Treatment of mice with IMC-18F1 significantly suppressed tumor growth. Moreover, immunohistochemical analysis of tumor xenografts collected from treated mice showed an increase of tumor cell apoptosis and a decrease in MAPK and AKT activation and cell proliferation [287];

(ii) The murine anti-human VEGFR-1 D16F7 mAb (mouse IgG1), which has a novel mechanism of action, as it interacts with a receptor site distinct from that involved in VEGF-A or PlGF binding and downregulates the signaling through the membrane receptor without affecting ligand binding [67]. Therefore, differently from other mAbs, which compete with the ligand for receptor binding, D16F7 does not increase the levels of free VEGF-A available for the activation of membrane VEGFR-2. Moreover, D16F7 leaves unaffected the sVEGFR-1 decoy function, as it neither affects sVEGFR-1 interaction with its ligands nor hampers the sVEGFR-1/VEGFR-2 inhibitory heterodimer formation [67,148,160]. D16F7 has shown antitumor efficacy in preclinical in vivo models against highly aggressive tumor types, such as glioblastoma and melanoma (see also Section 2.4 and Section 2.5) [145,148,160]. Its ability to recognize not only the human form of the VEGFR-1 but also the murine receptor has allowed the analysis of the effects of VEGFR-1 inhibition on tumor-associated microenvironment [148];

(iii) The anti-human VEGFR-1 KM1730 and KM1732 mAbs (mouse IgG1), which recognize different epitopes of the second Ig-like domain. Both mAbs prevented the binding of VEGF-A to VEGFR-1 and markedly inhibited monocyte migration after stimulation with the growth factor [288]. Moreover, treatment with KM1732 mAb of primary cell cultures derived from bone marrow samples collected from patients with AML resulted in significant inhibition of cell growth. This effect was more evident in cells with chromosomal abnormalities as compared to cells with a normal karyotype [289].

Regarding mAbs against the selective VEGFR-1 ligand PlGF, the humanized recombinant TB-403 (RO5323441) mAb, recognizes the receptor-binding site of the human and murine growth factor [290]. Significant tumor growth inhibition has been observed with TB-403 in medulloblastoma [53], hepatocellular carcinoma and renal cancer [291] xenograft models. In particular, three times weekly treatment with TB-403 of mice transplanted with the human medulloblastoma D283-MED or D341-MED cell lines resulted in a marked inhibition of the primary tumor growth and spinal metastasis formation [53]. Rizzo et al. instead reported growth inhibition ranging from 43 to 97% with twice-weekly dosing of TB-403 in mice transplanted with the renal carcinoma ACHN and Caki-1 cell lines and hepatocellular carcinoma Huh-7 cell line [291]. Another anti-human PlGF mAb (16D3) inhibited the growth and vascularization of colorectal or pancreatic cancer in xenograft models [112,215]. The anti-PlGF mAb 5D11D4 is directed against the murine PlGF of which inhibits the interaction with VEGFR-1 and with NRP-1 [292]. The 5D11D4 mAb inhibited tumor growth and metastasis formation in melanoma, cholangiocarcinoma, pancreatic, hepatocellular, and colon cancer in vivo models [112,113,114,215,292]. This effect was associated with reduced tumor infiltration by proangiogenic macrophages [215,292].

Experimental mAbs capable of specifically inhibiting human VEGFR-1 activation, for which preclinical evidence on their antitumor activity is available, are reported in Table 4.

The anticancer activity of IMC-18F1/icrucumab has been also evaluated in phase I and II clinical trials, the latter often investigating its potential in combination with other anticancer agents. In particular, the mAb combination with capecitabine has been tested in patients with unresectable, locally advanced, or metastatic breast cancer. However, this combined treatment failed to increase PFS, when compared to capecitabine alone or ramucirumab plus capecitabine [293]. The adverse events were similar in the combined treatment arms but higher compared to those observed when capecitabine was administered alone [293]. Similarly, no improvement in PFS was detected in patients with urothelial cancer carcinoma treated with the combination of IMC-18F1/icrucumab plus docetaxel, as second-line treatment of advanced/metastatic cancer, compared to the treatment with docetaxel alone or docetaxel plus ramucirumab [294]. In this study, adverse effects leading to drug discontinuation were more frequent in the ramucirumab containing arm than in the arm containing IMC-18F1/icrucumab [294]. Finally, the predetermined improvement in PFS was not observed in patients with metastatic colorectal cancer who received the combination of IMC-18F1/icrucumab with the modified FOLFOX-6 regimen (mFOLFOX), including folinic acid, 5-fluorouracil, and oxaliplatin, after progression during first-line therapy, in comparison with mFOLFOX-6 alone or mFOLFOX-6 plus the anti-VEGFR-2 mAb ramucirumab [295]. Discontinuation of study drugs due to treatment-emergent adverse effects occurred more frequently when chemotherapy was combined with ramucirumab (Table 5).

Concerning mAbs against VEGFR-1 ligands, a phase I/II clinical trial on the anti-PlGF mAb TB-403 with the purpose to assess the safety profile of graded doses of the mAb in pediatric subjects affected by relapsed or refractory medulloblastoma has been recently suspended. In previous phase I studies no serious adverse effects were reported [296,297,298].

Concerning new orally administered small-molecule RTK inhibitors, which target VEGFRs or a variety of RTKs including VEGFRs but lack selectivity toward VEGFR-1, several clinical trials are ongoing or have been recently completed with the aim of evaluating the safety, pharmacokinetics, pharmacodynamics, and clinical activity. Among investigational multi-targeted inhibitors with higher selectivity against VEGFRs, cediranib is active against the three VEGFRs with IC_50_ values in the range of low nanomolar concentrations but is more effective against VEGFR-2 [299]. Cediranib is currently evaluated in a large number of clinical trials against advanced/metastatic solid tumors as single agent or in combination therapies (e.g., NSCLC, mesothelioma, glioblastoma, RCC, ovarian, prostate, breast, endometrial, colorectal, pancreatic cancers, gastrointestinal stromal tumors, and AML). Fruquintinib has been found to have weak activity against RET, FGF receptors, and KIT kinases, whereas it inhibited more efficiently VEGFRs (VEGFR-3 >> VEGFR-2 > VEGFR-1) [300]. Fruquintinib has been mostly evaluated for gastro-intestinal tumors and NSCLC and was recently approved in China for the treatment of metastatic colorectal cancer. AEE788 is instead a dual inhibitor of epidermal growth factor and VEGF receptors and is currently under investigation in patients with recurrent glioblastoma, as single agent or in combination with everolimus. Another dual inhibitor is lucitanib, which targets FGF and VEGF receptors with higher inhibitory effect on VEGFR-2 than on VEGFR-1 kinase activity [301]. A total of thirty-two trials can be found in ClinicalTrials.gov where lucitanib is tested for solid tumors, breast cancer, NSCLC, SCLC, gastroesophageal reflux disease, and functional dyspepsia, with two of them including tumors with genetic alterations of the FGF receptor.

Among agents inhibiting a variety of receptor kinases comprising VEGFRs, sitravatinib (MGCD516) is tested in phase I/II studies as monotherapy for advanced solid tumors, locally advanced/unresectable soft tissue sarcomas in neo-adjuvant setting, and triple-negative metastatic breast cancer. Moreover, sitravatinib is evaluated in combination with immune checkpoint inhibitors (i.e., the anti-PD1 mAbs nivolumab or tislelizumab) for resectable squamous cell carcinoma of the oral cavity, advanced/metastatic urothelial, kidney, gastric, or hepatocellular carcinomas, based on its ability to modulate innate and adaptive immunity by targeting the tumor microenvironment [302]. A phase III trial is also comparing sitravatinib plus nivolumab with docetaxel chemotherapy for advanced non-squamous NSCLC. Dovitinib is a multi-kinase inhibitor with the ability to target FGF and PDGF receptors, KIT and RET kinases as well as VEGFRs, with higher inhibitory effects toward VEGFR-3 and VEGFR-1 than toward VEGFR-2 (IC_50_ values 2.5 nm, 3.2 nM and 20 nM, respectively) [301]. This inhibitor is investigated, as single agent or as combined treatment, in 48 clinical trials against multiple myeloma and a large variety of solid tumors.

## 4. Conclusions

A recurrent feature of highly aggressive metastatic solid tumors is represented by an augmented expression and activation of VEGFR-1, in association with increased levels of its ligands VEGF-A, VEGF-B, and PlGF.

Based on the crucial role of blood vessel formation in cancer progression, a number of antiangiogenic agents have been developed and approved. Inhibition of VEGF-A and its signaling through VEGFR-2, a receptor also involved in physiological angiogenesis, causes important adverse effects such as hypertension, proteinuria, bleeding, thromboembolism, delay in wound healing, and gastrointestinal perforation. Besides, a controversy has arisen concerning the application of therapies exclusively targeting blood vessels: the disruption of endothelial cell barrier caused by these agents might facilitate tumor cell extravasation. Moreover, the hypoxic condition induced by antiangiogenic treatments directly contributes to tumor cells EMT; metabolism shift; ECM invasion; and, paradoxically, may favor metastasis [303]. Conversely, the neutralization of VEGFR-1 specific ligands, i.e., VEGF-B and PlGF, and the selective blockade of VEGFR-1 activation, represents a promising strategy to specifically counteract tumor-associated angiogenesis as well as malignant processes not directly related to new blood vessels formation. Indeed, inhibition of VEGFR-1 signaling also reduces tumor cell survival and invasiveness, counteracts the mobilization of myeloid progenitors and prevents tumor infiltration by M2 protumoral macrophages.

In this context, the D16F7 mAb is a promising tool to specifically and non-competitively interfere with VEGFR-1 signaling in the tumor. The ligand-binding site is not occupied by the mAb and, therefore, the VEGF-A, VEGF-B or PlGF binding is not inhibited. Based on this property, the overall quantity of VEGF-A available to stimulate VEGFR-2 does not change and the antiangiogenic activity of the soluble receptor isoform sVEGFR-1 remains unaffected [67]. Conversely, other experimental mAbs designed to inhibit human VEGFR-1 signaling, like IMC-18F1/icrucumab, KM1730, or KM1732, possess a competitive mechanism of action, by inhibiting VEGF-A, VEGF-B, or PlGF binding to VEGFR-1. Competitive VEGFR-1 inhibitors, by antagonizing ligand binding, may increase in the ECM the free VEGF-A available for VEGFR-2 activation and this may reduce the overall efficacy of the treatment. Moreover, competitive mAbs, through their binding to sVEGFR-1, may also counteract its antiangiogenic effects. All these factors might have contributed to the reported failure of clinical trials observed with the competitive inhibitor IMC-18F1/icrucumab (see Table 5).

D16F7 mAb efficacy in monotherapy has already been demonstrated in in vivo preclinical models of highly aggressive tumors, showing significant inhibition of glioblastoma and melanoma growth, invasiveness and migration, impairment of endothelial cells chemotaxis and tumor-associated angiogenesis, as well as reduced myeloid progenitor mobilization and tumor infiltration by monocytes/macrophages [145,148,160]. These data encourage further studies aimed at testing D16F7 effectiveness in metastatic tumor models as a single agent or in combination with antiangiogenic agents targeting VEGF-A/VEGFR-2 signaling or immune checkpoint inhibitors.

## Figures and Tables

**Table 1:** Experimental evidence of the role of VEGFR-1 and its ligands in metastasis formation or tumor invasiveness of lung, liver, kidney, bone and pancreatic cancers, glioblastoma, and melanoma.

Tumor Type	Metastatic Sites	VEGFR-1 Ligands	VEGFR-1
Lung cancer			
NSCLC	Bone, brain, liver, and adrenal glands	- High VEGF-A expression in tumor specimens is associated with shorter survival time [83]	- High VEGFR-1 and VEGFR-2 expression in tumor specimens have been regarded as prognostic markers [83]
		- VEGF-A downregulation, through HIF-1α knockdown, reduces the invasiveness of a human lung carcinoma cell line [85]	- PlGF-mediated activation of VEGFR-1 is involved in macrophages polarization to TAMs [78]
		- VEGF-A expression is modulated by several miRNAs whose expression is decreased in tumor cell lines and tissue samples [86,87,88,89,90,91]	- VEGFR-1 is required for the infiltration by BMDCs that stimulate the growth of metastatic nodules [103]
		- IL-17 stimulates VEGF-A expression and angiogenesis by activating the STAT3/GIV signaling pathway [92]	
		- Tumor-derived VEGF-A promotes its own secretion, through PI3K/AKT, STAT3, RAS/ERK signaling pathways [81]	
		- VEGF-A165 regulates the expression of sVEGFR1-i13 splice variant of sVEGFR-1 through SOX2 and SRSF2 proteins [93]	
		- High PlGF levels in NSCLC specimens from patients with distal metastases are associated with poor survival, increased tumor invasiveness and enhanced MMP9 expression [95]	
		- High PlGF levels correlate with an increased ratio between the proangiogenic isoform VEGF-A165 and the antiangiogenic isoform VEGF-A165b, through induction of the splicing regulatory factor SRp40 [96]	
		- PlGF secreted by tumor cells induces macrophages polarization to TAMs [78]	
		- High VEGF-B expression is associated with poor survival in patients with squamous cell carcinoma [69]	
SCLC	Liver	- VEGF-A levels correlate with microvessel density [98]	-
Mesothelioma	Bone and other contiguous organs (spinal cord, pericardium, and contralateral lung)	- PlGF is overexpressed in malignant mesothelioma specimens and cell lines [100,101]	- VEGFR-1 is overexpressed in patients and in malignant mesothelioma cell lines [100,101]
Liver			
Hepatocellular carcinoma	Lung, bone, lymph nodes, and adrenal glands	- High circulating VEGF-A correlates with tumor angiogenesis and reduced survival [106,107,108,109]	- VEGFR-1 activation by VEGF-B induces tumor cell migration and invasion by activating MMP9 and increasing the expression of the EMT regulator Snail; high coexpression of VEGFR-1 and MMP9 act as prognostic marker [115,116]
		- The VEGF-B186 is frequently upregulated compared to the VEGF-B167 and its expression correlates with cancer growth and invasiveness [110]	
		- VEGF-A secretion is reduced by miR-199a-3p [111] - PlGF in vivo blockade results in normalization of tumor-associated vessels, reduced tumor nodule formation and increased survival [112,113] - VEGF-B favors molecular and morphological alterations of EMT [115]	- VEGFR-1 and VEGFR-2 expression on endothelial cells is reduced by miR-199a-3p [111] - VEGFR-1 is required for the vascularization of liver metastases from renal cell carcinoma [124]
Cholangiocarcinoma	Lymph nodes, liver, and peritoneum	- PlGF inhibition, by treatment with the 5D11D4 mAb decreases tumor burden and infiltration by protumoral M2 cells in chemically-induced hepatocellular and cholangiocarcinoma in vivo models [114] - VEGF-A expression is associated with angiogenesis, metastasis and tumor recurrence [119] - High VEGF-A levels in tumor tissues correlate with a marked decrease of miR-101, a miRNA capable of downregulating VEGF-A transcript [120]	
Gallbladder	Lymph nodes, liver, and peritoneum	- High VEGF-A levels in patients with gallbladder cancer promote angiogenesis and tumor cell proliferation/invasion [121] - VEGF-A expression correlates with poor prognosis [122] - PlGF is overexpressed and stimulates EMT through c-MYC upregulation and consequent induction of miR-19a [123] - Patients with high PlGF and miR-19a levels in the tumor show a shorter overall survival (OS) than patients with low expression [123]	-
Renal cell carcinoma	Lung, bone, lymph nodes, liver, adrenal gland, and brain	- In clear-cell renal cell carcinoma VEGF-A overexpression is due to mutations or epigenetic inactivation of the *VHL* gene, in turn responsible for HIF-1α accumulation [128,129].	- VEGFR-1 and VEGF-B expression is higher in tumor tissues compared to normal renal tissues [137]
		- 2578C/A, +936C/T and +405G/C single nucleotide polymorphisms in the *VEGF* gene correlate with elevated risk of developing renal cell carcinoma [133,134] - VEGF-A expression is suppressed by Dicer, an endoribonuclease downregulated in clear cell renal cell carcinoma [135] - VEGF-A protein levels are associated with tumor size, tumor grade, and metastasis at diagnosis [136] - PlGF plasma concentrations increase after treatment with the multi-targeted RTK inhibitor sunitinib [139]	- Tumor infiltration by bone-marrow derived myeloid cells expressing VEGFR-1 contributes to neovessel formation and immune escape [138]
Glioblastoma	Only in 0.4–0.5% of all cases extra-cranial metastases in the spinal cord, vertebrae, lung, liver, and lymph nodes	- VEGF-A and PlGF binding to VEGFR-1 enhances migration, and ECM invasion in glioblastoma cell lines through VEGFR-1 phosphorylation at Tyr 1213 and ERK1/2 phosphorylation [145]	- In high-grade glioma specimens, VEGFR-1 is expressed at significantly higher levels than in low-grade glioma [146]
		- VEGF-A- and PlGF-mediated activation of VEGFR-1 stimulates tumor growth and angiogenesis in an in vivo preclinical glioma model [148]	- Glioblastoma cells (cell lines and tumor specimens from patients) and primary cultures of glioblastoma stem cells express VEGFR-1 [145]
		- VEGF-A expression is higher in glioblastoma than in low-grade gliomas and plays a role in the switch to a highly vascularized tumor [31] - GAMs contribute to tumor progression and resistance to anti-VEGF-A therapy [149,150,151,152,153]	- High expression of VEGFR-1 confers metastatic traits via modulation of SHH signaling pathway [147] - Low sVEGFR-1/VEGF-A ratio correlates with high tumor aggressiveness [31] - In surgical specimens from glioblastoma patients, VEGFR-1 is expressed in GAMs [154]
Melanoma	Skin, lung, brain, liver, bone, and intestine	- Expression of PlGF and VEGF-A is associated with enhanced tumor cell proliferation, migration and ECM invasion [157,158]	- Expression of VEGFR-1 correlates with tumor cell proliferation, migration and ECM invasion [157]
		- VEGF-A and PlGF release is more frequent among cell lines derived from metastatic melanoma than from primary tumor [157] - Cell lines secreting VEGF-A and expressing VEGFR-1 spontaneously migrate through matrigel-coated filters [159] - PlGF induces tumor growth, vascularization, and metastases in a preclinical in vivo model [162] - PlGF induces resistance to temozolomide through NF-κB activation [163]	- The *sVEGFR-1/VEGFR-1* transcript ratio decreases in cutaneous metastases compared to primary tumors, due to decrease of *sVEGFR-1* mRNA levels [36] - Upregulation of VEGFR-1 contributes to resistance toward BRAFi [161]
Bone			
Osteosarcoma	Lungs, other bones, and lymph nodes	- Increased VEGF-A serum levels are associated with enhanced vessel density in the tumor and decreased survival [170,171,172] - *VEGF-A* gene polymorphisms +936C/T and –634 G/C are associated with the risk of developing osteosarcoma [173] - VEGF-A expression is promoted by HIF-1α in hypoxic conditions in osteosarcoma cells; HIF-1 and VEGF-A knockdown decreases the invasive potential of osteosarcoma cells [174] - miR-134 binds to *VEGF-A* transcript and attenuates tumor growth and neovessel formation [177] - miR-1 inhibits *VEGF-A* expression at the post-transcriptional level, by binding to its mRNA 3′-UTR [178]	- A constitutive activation of an autocrine VEGF-A/VEGFR-1 signaling pathway is reported in highly aggressive osteosarcoma [175] - VEGF-A/VEGFR-1 signaling is a target of miR-134, which binds to VEGFR-1 transcript and attenuates tumor growth and neovessel formation [177].
Ewing sarcoma	Lungs, bone, and bone marrow	- High levels of the VEGF-A165 isoform contribute to stimulate the osteolytic process [179,180] - VEGF-A upregulates RANKL and increases the recruitment of TAMs in the tumor [181]	-
Chondrosarcoma	Lung and skeleton	- VEGF-A expression correlates with adiponectin expression and tumor stage [182] - VEGF-A expression is inhibited by miR-452, which in turn is downregulated by the high expression of WISP-3, stimulating angiogenesis [183]	-
Pancreas	Liver, lung, and peritoneum; rarely bone, adrenal gland, and distant lymph nodes	- VEGF-A is expressed in ductal epithelial tumor cells, but not in ductal cells of non-transformed pancreas or chronic pancreatitis [187,188] - VEGF-A influences pancreatic tumor cells glucose metabolism: it enhances glycolysis via HIF-1α upregulation and NRP-1 involvement [189] - Increased levels of PlGF are reported in obesity-associated pancreatic cancer patients [77] - VEGF-A mRNA and protein are detected in human ductal pancreatic carcinoma cell lines [188]	- Ablation of the VEGFR-1 signaling in pancreatic ductal adenocarcinoma murine models prevents obesity-induced tumor progression [77] - VEGFR-1 and VEGFR-2 are expressed in 29% and 43% of pancreatic carcinoma tissues, respectively [188] - VEGFR-1 expression is observed in human ductal pancreatic carcinoma cell lines [188] - VEGFR-1 and VEGFR-2 coexpression has been recognized as a poor prognostic factor [190] - VEGFR-1 and VEGFR-3 expression is significantly higher in tumor cells and tumor-associated endothelial cells, while VEGFR-2 is detected only in tumor cells [191]

**Table 2:** Experimental evidence of the involvement of VEGFR-1 and its ligands in the metastatic spreading of cancers affecting the gastrointestinal tract.

Tumor Type	Metastatic Sites	VEGFR-1 Ligands	VEGFR-1
Oral	Hypopharynx, tongue, and lymph nodes	- VEGF-A overexpression correlates with poor prognosis [194,195] - Increased expression of VEGF-A positively correlates with disease recurrence and lymph node metastases in gingival cancer [196]	- VEGFR-1 signaling stimulates bone invasion, by promoting differentiation and activation of preosteoclasts [197]
Esophageal	Liver, lymph nodes, lung, bone, and brain	- VEGF-A overexpression increases the risk of distant metastases and shorter OS [196] - The rs2010963 genetic polymorphism in *VEGF-A* correlates with worse OS [202]	- VEGFR-1 expression inversely correlates with patients’ survival [201]
Gastric	Liver, peritoneum, lung, and bone	- High VEGF-A levels are associated with increased CRMP4 expression [207] - High VEGF-A levels are detected in both serum and plasma of gastric cancer patients [208] - PlGF promotes cell proliferation and chemotaxis through activation of PI3K/AKT and p38 MAPK signaling pathways [209]	- VEGFR-1 is associated with the formation of hematogenous metastases if co-detected with isolated tumor cells [205]
Colorectal	Liver, peritoneum, lung, bone, and brain	- VEGF-A and VEGF-B, by stimulating VEGFR-1, induce downstream ERK-1/2 and JNK MAPK signaling pathways, increasing cell migration, invasion and proliferation [212] - PlGF expression is associated with higher cell invasion/migration [214] - PlGF is more expressed in patients with lymph node metastases, than in patients without metastases, and correlates with poor prognosis [216] - An intracrine VEGF-A/VEGFR-1 signaling mediates cancer cell survival [218]	- VEGFR-1 expression and activation promote phenotypic changes associated with tumor progression and metastases [212] - Stimulation of VEGFR-1 by VEGF-A increases cell migration and tyrosine phosphorylation of FAK, paxillin, and p130cas [213] - VEGFR-1 activation by PlGF induces invasion and migration, due to phosphorylation of p38 MAPK and upregulation of MMP9 expression [214] - VEGFR-1 blockade by the iVR1 peptide markedly inhibits tumor growth and recruitment of monocyte/macrophages at the tumor site in syngeneic and xenograft colorectal cancer models [215] - VEGFR-1 signaling, activated by intracrine VEGF-A, regulates cell migration through modulation of FAK activity [219] - VEGFR-1 expressing myeloid cells are crucial for tumor metastatic growth and angiogenesis in the liver [220]

**Table 3:** Experimental evidence of VEGFR-1 and its ligands in the metastatic evolution of sex-specific cancers.

Tumor Type	Metastatic Sites	VEGFR-1 Ligands	VEGFR-1
Breast	Bone, lung, liver, and brain	- The VEGF-A-stimulated-PKC pathway induces a pre-metastatic destabilization of pulmonary tight junctions, promoting vessel permeability and extravasation [225] - VEGF-A is involved in breast cancer liver metastases [227] - MiR-126 overexpression in human breast cancer inhibits cell proliferation by reducing VEGF-A levels [228] - Overexpression of COX-2-induced miR526b and miR655 in tumor cells upregulates VEGF-A [229] - VEGF-A signaling pathway and MMPs expression are positively regulated by cystathionine -γ-lyase, a key enzyme in the biosynthesis of the proangiogenic H_2_S [232] - PlGF stimulates cancer cell motility through activation of intracellular signaling cascades, including ERK1/2, and cytoskeletal remodeling [233] - PlGF favors CD34^+^ differentiation into tumor-mobilized bone marrow-derived CD11b^+^ myeloid cells [235]	- In breast cancer cells overexpression of COX-2-induced miR526b and miR655 results in upregulation of VEGFR-1 [229] - VEGFR-1 signaling, due to PlGF interaction, is associated with obesity-induced breast cancer progression [77]
Ovary	Peritoneum, liver, lymph nodes, bone, and brain	- High expression of VEGF-A is considered a prognostic factor [239] - VEGF-A contributes to the recruitment and activation of myeloid-derived suppressor cells [242] - PlGF is overexpressed and increases MMP7 expression via downregulation of miR-543 [243] - PlGF overexpression increases ZEB2 expression in a p38 MAPK-dependent manner [245]	- sVEGFR-1 induces cell death in a murine model of ovarian cancer [246]
Cervix	Lung, bone, liver, and brain	- Serum VEGF-A is a promising prognostic biomarker [249] - VEGF-A is highly expressed in specimens from patients with post-radiotherapy relapsed/persistent cancer [250] - VEGF-A is coexpressed with a metabolism-related protein (MCT4 isoform) [251] - PlGF induces EMT, promoting migration and metastases, through activation of the ERK/MAPK signaling pathway [252] - VEGF-A in culture supernatants from cervical cancer cell lines induces an M2-like phenotype [254]	- High VEGFR-1 expression is associated with distant metastases, poor OS and PFS [248] - VEGFR-1 is highly expressed in specimens from patients with post-radiotherapy relapsed/persistent cancer [250]
Prostate	Bone, lymph nodes, lung, liver, brain	- VEGF-A expression and tumor invasiveness are favored by an acidic environment, through increased MMP9 secretion [256] - VEGF-A expression is higher in prostate cancer samples than in BPH and HGPIN samples [259] - VEGF-A decreases the expression of miR-130b and abrogates its antiangiogenic effect [261] - VEGF-A is upregulated by androgens [262] - VEGF-A inhibits the maturation and activity of DCs [264]	- VEGFR-1 expression is higher in prostate cancer samples than in BPH and HGPIN samples [259]

**Table 4:** Experimental mAbs designed to inhibit human VEGFR-1 signaling.

Name	Target	Mechanism of Action	In Vivo Antitumor Activity	Ref.
IMC-18F1/ Icrucumab	Human VEGFR-1	Competitive: prevention of VEGF-A, VEGF-B and PlGF binding to VEGFR-1	Suppression of tumor growth, by increasing apoptosis and decreasing cell proliferation in human breast cancer xenografts	[287]
D16F7	Human VEGFR-1	Non-competitive: down-modulation of VEGFR-1 signaling without inhibition of VEGF-A or PlGF binding	Decrease of tumor growth and tumor-associated angiogenesis and increase of animal survival in heterotopic and orthotopic glioblastoma murine models	[148]
			Inhibition of tumor growth, tumor infiltration by monocytes/macrophages and bone invasion by cancer cells in a syngeneic murine melanoma model	[160]
KM1730/ KM1732	Human VEGFR-1	Competitive: blockade of VEGFR-1 interaction with VEGF-A	-	[288,289]
TB-403 (RO5323441)	Human PlGF	Interaction with the receptor-binding site of PlGF	Inhibition of primary tumor growth and spinal metastases in medulloblastoma murine models	[53]
			Tumor growth inhibition in hepatocarcinoma and renal cancer murine models	[291]
16D3	Human PlGF	Interaction with the receptor-binding site of PlGF	Tumor growth inhibition in colorectal and pancreatic cancer xenograft models	[112,215]

**Table 5:** Clinical trials involving selective VEGFR-1 signaling inhibitors.

ClinicalTrials.gov Identifier Code	Phase and Status	Agent	Combined Drugs and Comparators	Tumor Type	Ref.
NCT01234402	II, Completed	IMC-18F1/ Icrucumab	Icrucumab + capecitabine *Vs* Ramucirumab + capecitabine or capecitabine	Breast cancer (previously treated, stage III/IV)	[293]
NCT01282463	II, Completed	IMC-18F1/ Icrucumab	Icrucumab + docetaxel *Vs* Docetaxel + ramucirumab or docetaxel	Locally advanced or metastatic carcinoma of the urinary tract	[294]
NCT01111604	II, completed	IMC-18F1/ Icrucumab	Icrucumab + mFOLFOX-6 *Vs* mFOLFOX-6 + ramucirumab or mFOLFOX-6	Metastatic colorectal cancer after progression during first-line chemotherapy	[295]
NCT00782002	I, Completed	IMC-18F1/ Icrucumab	-	Advanced/ refractory solid tumors	www.ClinicalTrials.gov

## Abbreviations

AKT	Protein Kinase B
BMDCs	Bone marrow-derived cells
BPH	Benign prostatic hyperplasia
BRAFi	BRAF inhibitors
ccRCC	Clear-cell renal cell carcinoma
CNS	Central nervous system
COX	Cyclooxygenase
CRMP4	Collapsin response mediator protein family 4
CSE	Cystathionine-γ-lyase
DCs	Dendritic cells
ECM	Extracellular matrix
EMT	Epithelial/mesenchimal transition
ERK	Extracellular signal-regulated kinase
FAK	Focal adhesion kinase
FGF	Fibroblast growth factor
GAMs	Glioblastoma-associated microglia/macrophages
GIV	Gα–Interacting Vesicle-associated protein
H2S	Hydrogen sulfide
HGPIN	High-grade prostate intraepithelial neoplasia
HIF	Hypoxia-inducible factor
HUVECs	Human umbilical vein endothelial cells
Ig	Immunoglobulin
IL	Interleukin
mAb	Monoclonal antibody
MAMs	Metastasis associated macrophages
MAPK	Mitogen-activated protein kinases
MCT	Monocarboxylate transporters
miRNA	MicroRNA
MMP	Matrix metalloproteinase
NFkB	Nuclear factor -kB
NK	Natural killer
NRP	Neuropilin
NSCLS	Non small-cell lung cancer
ORR	Overall response rate
OS	Overall survival
OSCC	Oral squamous cell carcinomas
PDGF	Platelet derived growth factor
PFS	Progression-free survival
PI3K	Phosphatidylinositol-3-kinase
PKC	Protein kinase C
PlGF	Placenta growth factor
RANK	Receptor activator of nuclear factor kappa-Β
RANKL	Receptor activator of nuclear factor kappa-Β ligand
RCC	Renal cell carcinoma
RTKs	Receptor tyrosine kinases
SCLS	Small-cell lung cancer
SHH	Sonic Hedgehog Homolog
shRNA	Short hairpin RNA
STAT3	Signal transducer and activator of transcription 3
sVEGFR	Soluble vascular endothelial growth factor receptor
TAMs	Tumor associated macrophages
TCGA	The Cancer Genome Atlas
TK	Tyrosine kinase
Tregs	Regulatory T cells
VEGF	Vascular endothelial growth factor
VEGFR	Vascular endothelial growth factor receptor
VHL	von Hippel–Lindau

## References

[1] Siveen K.S., Prabhu K., Krishnankutty R., Kuttikrishnan S., Tsakou M., Alali F.Q., Dermime S., Mohammad R.M., Uddin S. (2017). Vascular Endothelial Growth Factor (VEGF) Signaling in Tumour Vascularization: Potential and Challenges. Curr. Vasc. Pharmacol..

[2] Karaman S., Leppänen V.M., Alitalo K. (2018). Vascular endothelial growth factor signaling in development and diseas. Development.

[3] De Aguiar R.B., De Moraes J.Z. (2019). Exploring the Immunological Mechanisms Underlying the Anti-vascular Endothelial Growth Factor Activity in Tumors. Front. Immunol..

[4] Ferrara N. (2004). Vascular endothelial growth factor as a target for anticancer therapy. Oncologist.

[5] Maglione D., Guerriero V., Viglietto G., Delli-Bovi P., Persico M.G. (1991). Isolation of a human placenta cDNA coding for a protein related to the vascular permeability factor. Proc. Natl. Acad. Sci. USA.

[6] Cao Y., Ji W.R., Qi P., Rosin A., Cao Y. (1997). Placenta growth factor: Identification and characterization of a novel isoform generated by RNA alternative splicing. Biochem. Biophys. Res. Commun..

[7] Carmeliet P., Moons L., Luttun A., Vincenti V., Compernolle V., De Mol M., Wu Y., Bono F., Devy L., Beck H. (2001). Synergism between vascular endothelial growth factor and placental growth factor contributes to angiogenesis and plasma extravasation in pathological conditions. Nat. Med..

[8] Fischer C., Mazzone M., Jonckx B., Carmeliet P. (2008). FLT1 and its ligands VEGFB and PlGF: Drug targets for anti-angiogenic therapy?. Nat. Rev. Cancer.

[9] Mould A.W., Tonks I.D., Cahill M.M., Pettit A.R., Thomas R., Hayward N.K., Kay G.F. (2003). Vegfb gene knockout mice display reduced pathology and synovial angiogenesis in both antigen-induced and collagen-induced models of arthritis. Arthritis Rheum..

[10] Li X. (2010). VEGF-B: A thing of beauty. Cell Res..

[11] Peach C.J., Mignone V.W., Arruda M.A., Alcobia D.C., Hill S.J., Kilpatrick L.E., Woolard J. (2018). Molecular Pharmacology of VEGF-A Isoforms: Binding and Signalling at VEGFR2. Int. J. Mol. Sci..

[12] Joukov V., Sorsa T., Kumar V., Jeltsch M., Claesson-Welsh L., Cao Y., Saksela O., Kalkkinen N., Alitalo K. (1997). Proteolytic processing regulates receptor specificity and activity of VEGF-C. EMBO J..

[13] Stacker S.A., Stenvers K., Caesar C., Vitali A., Domagala T., Nice E., Roufail S., Simpson R.J., Moritz R., Karpanen T. (1999). Biosynthesis of vascular endothelial growth factor-D involves proteolytic processing which generates non-covalent homodimers. J. Biol. Chem..

[14] Lymboussaki A., Olofsson B., Eriksson U., Alitalo K. (1999). Vascular endothelial growth factor (VEGF) and VEGF-C show overlapping binding sites in embryonic endothelia and distinct sites in differentiated adult endothelia. Circ. Res..

[15] Karkkainen M.J., Haiko P., Sainio K., Partanen J., Taipale J., Petrova T.V., Jeltsch M., Jackson D.G., Talikka M., Rauvala H. (2004). Vascular endothelial growth factor C is required for sprouting of the first lymphatic vessels from embryonic veins. Nat. Immunol..

[16] Rauniyar K., Jha S.K., Jeltsch M. (2018). Biology of Vascular Endothelial Growth Factor C in the Morphogenesis of Lymphatic Vessels. Front. Bioeng. Biotechnol..

[17] Stacker S.A., Caesar C., Baldwin M.E., Thornton G.E., Williams R.A., Prevo R., Jackson D.G., Nishikawa S., Kubo H., Achen M.G. (2001). VEGF-D promotes the metastatic spread of tumor cells via the lymphatics. Nat. Med..

[18] Matthews W., Jordan C.T., Wiegand G.W., Pardoll D., Lemischka I.R. (1991). A receptor tyrosine kinase specific to hematopoietic stem and progenitor cell-enriched populations. Cell.

[19] Terman B.I., Carrion M.E., Kovacs E., Rasmussen B.A., Eddy R.L., Shows T.B. (1991). Identification of a new endothelial cell growth factor receptor tyrosine kinase. Oncogene.

[20] Simons M., Gordon E., Claesson-Welsh L. (2016). Mechanisms and regulation of endothelial VEGF receptor signalling. Nat. Rev. Mol. Cell Biol..

[21] Park J.E., Chen H.H., Winer J., Houck K.A., Ferrara N. (1994). Placenta growth factor. Potentiation of vascular endothelial growth factor bioactivity, in vitro and in vivo, and high affinity binding to Flt-1 but not to Flk-1/KDR. J. Biol. Chem..

[22] Olofsson B., Korpelainen E., Pepper M.S., Mandriota S.J., Aase K., Kumar V., Gunji Y., Jeltsch M.M., Shibuya M., Alitalo K. (1998). Vascular endothelial growth factor B (VEGF-B) binds to VEGF receptor-1 and regulates plasminogen activator activity in endothelial cells. Proc. Natl. Acad. Sci. USA.

[23] Luttun A., Tjwa M., Moons L., Wu Y., Angelillo-Scherrer A., Liao F., Nagy J.A., Hooper A., Priller J., De Klerck B. (2002). Revascularization of ischemic tissues by PlGF treatment, and inhibition of tumor angiogenesis, arthritis and atherosclerosis by anti-Flt1. Nat. Med..

[24] Hiratsuka S., Nakao K., Nakamura K., Katsuki M., Maru Y., Shibuya M. (2005). Membrane fixation of vascular endothelial growth factor receptor 1 ligand-binding domain is important for vasculogenesis and angiogenesis in mice. Mol. Cell Biol..

[25] Bae D.G., Kim T.D., Li G., Yoon W.H., Chae C.B. (2005). Anti-flt1 peptide, a vascular endothelial growth factor receptor 1-specific hexapeptide, inhibits tumor growth and metastasis. Clin. Cancer Res..

[26] Kendall R.L., Thomas K.A. (1993). Inhibition of vascular endothelial cell growth factor activity by an endogenously encoded soluble receptor. Proc. Natl. Acad. Sci. USA.

[27] Ambati B.K., Nozaki M., Singh N., Takeda A., Jani P.D., Suthar T., Albuquerque R.J., Richter E., Sakurai E., Newcomb M.T. (2006). Corneal avascularity is due to soluble VEGF receptor-1. Nature.

[28] Nominato L.F., Dias A.C., Dias L.C., Fantucci M.Z., Mendes da Silva L.E.C., Murashima A.A., Rocha E.M. (2018). Prevention of Corneal Neovascularization by Adenovirus Encoding Human Vascular Endothelial Growth Factor Soluble Receptor (s-VEGFR1) in Lacrimal Gland. Invest Ophthalmol Vis. Sci..

[29] Koga K., Osuga Y., Yoshino O., Hirota Y., Ruimeng X., Hirata T., Takeda S., Yano T., Tsutsumi O., Taketani Y. (2003). Elevated serum soluble vascular endothelial growth factor receptor 1 (sVEGFR-1) levels in women with preeclampsia. J. Clin. Endocrinol. Metab..

[30] Maynard S.E., Min J.Y., Merchan J., Lim K.H., Li J., Mondal S., Libermann T.A., Morgan J.P., Sellke F.W., Stillman I.E. (2003). Excess placental soluble fms-like tyrosine kinase 1 (sFlt1) may contribute to endothelial dysfunction, hypertension, and proteinuria in preeclampsia. J. Clin. Invest..

[31] Lamszus K., Ulbricht U., Matschke J., Brockmann M.A., Fillbrandt R., Westphal M. (2003). Levels of soluble vascular endothelial growth factor (VEGF) receptor 1 in astrocytic tumors and its relation to malignancy, vascularity, and VEGF-A. Clin. Cancer Res..

[32] Aref S., El Sherbiny M., Goda T., Fouda M., Al Askalany H., Abdalla D. (2005). Soluble VEGF/sFLt1 ratio is an independent predictor of AML patient out come. Hematology.

[33] Bando H., Weich H.A., Brokelmann M., Horiguchi S., Funata N., Ogawa T., Toi M. (2005). Association between intratumoral free and total VEGF, soluble VEGFR-1, VEGFR-2 and prognosis in breast cancer. Br. J. Cancer.

[34] Chang Y.T., Chang M.C., Wei S.C., Tien Y.W., Hsu C., Liang P.C., Tsao P.N., Jan I.S., Wong J.M. (2008). Serum vascular endothelial growth factor/soluble vascular endothelial growth factor receptor 1 ratio is an independent prognostic marker in pancreatic cancer. Pancreas.

[35] Nagaoka S., Yoshida T., Akiyoshi J., Akiba T., Hisamoto T., Yoshida Y., Abe M., Koga H., Toirimura T., Ueno T. (2010). The ratio of serum placenta growth factor to soluble vascular endothelial growth factor receptor-1 predicts the prognosis of hepatocellular carcinoma. Oncol. Rep..

[36] Ruffini F., Failla C.M., Orecchia A., Bani M.R., Dorio A.S., Fortes C., Zambruno G., Graziani G., Giavazzi R., D’Atri S. (2011). Expression of the soluble vascular endothelial growth factor receptor-1 in cutaneous melanoma: Role in tumour progression. Br. J. Dermatol..

[37] Takano S., Ishikawa E., Matsuda M., Sakamoto N., Akutsu H., Yamamoto T., Matsumura A. (2017). The anti-angiogenic role of soluble-form VEGF receptor in malignant gliomas. Int.J. Oncol..

[38] Orecchia A., Lacal P.M., Schietroma C., Morea V., Zambruno G., Failla C.M. (2003). Vascular endothelial growth factor receptor-1 is deposited in the extracellular matrix by endothelial cells and is a ligand for the alpha 5 beta 1 integrin. J. Cell. Sci..

[39] Orecchia A., Mettouchi A., Uva P., Simon G.C., Arcelli D., Avitabile S., Ragone G., Meneguzzi G., Pfenninger K.H., Zambruno G. (2014). Endothelial cell adhesion to soluble vascular endothelial growth factor receptor-1 triggers a cell dynamic and angiogenic phenotype. FASEB J..

[40] Abou Faycal C., Brambilla E., Agorreta J., Lepeltier N., Jacquet T., Lemaître N., Emadali A., Lucas A., Lacal P.M., Montuenga L. (2018). The sVEGFR1-i13 splice variant regulates a β1 integrin/VEGFR autocrine loop involved in the progression and the response to anti-angiogenic therapies of squamous cell lung carcinoma. Br. J. Cancer.

[41] Stacker S.A., Achen M.G. (2018). Emerging Roles for VEGF-D in Human Disease. Biomolecules.

[42] Su J.L., Yen C.J., Chen P.S., Chuang S.E., Hong C.C., Kuo I.H., Chen H.Y., Hung M.C., Kuo M.L. (2007). The role of the VEGF-C/VEGFR-3 axis in cancer progression. Br. J. Cancer.

[43] Karnezis T., Shayan R., Caesar C., Roufail S., Harris N.C., Ardipradja K., Zhang Y.F., Williams S.P., Farnsworth R.H., Chai M.G. (2012). VEGF-D promotes tumor metastasis by regulating prostaglandins produced by the collecting lymphatic endothelium. Cancer Cell.

[44] Sarabipour S., Ballmer-Hofer K., Hristova K. (2016). VEGFR-2 conformational switch in response to ligand binding. eLife.

[45] Tvorogov D., Anisimov A., Zheng W., Leppänen V.M., Tammela T., Laurinavicius S., Holnthoner W., Heloterä H., Holopainen T., Jeltsch M. (2010). Effective suppression of vascular network formation by combination of antibodies blocking VEGFR ligand binding and receptor dimerization. Cancer Cell..

[46] Kendrew J., Eberlein C., Hedberg B., McDaid K., Smith N.R., Weir H.M., Wedge S.R., Blakey D.C., Foltz I., Zhou J. (2011). An antibody targeted to VEGFR-2 Ig domains 4-7 inhibits VEGFR-2 activation and VEGFR-2-dependent angiogenesis without affecting ligand binding. Mol. Cancer Ther..

[47] Becker P.M., Waltenberger J., Yachechko R., Mirzapoiazova T., Sham J.S., Lee C.G., Elias J.A., Verin A.D. (2005). Neuropilin-1 regulates vascular endothelial growth factor-mediated endothelial permeability. Circ. Res..

[48] Fantin A., Lampropoulou A., Senatore V., Brash J.T., Prahst C., Lange C.A., Liyanage S.E., Raimondi C., Bainbridge J.W., Augustin H.G. (2017). VEGF165-induced vascular permeability requires NRP1 for ABL-mediated SRC family kinase activation. J. Exp. Med..

[49] Soker S., Takashima S., Miao H.Q., Neufeld G., Klagsbrun M. (1998). Neuropilin-1 is expressed by endothelial and tumor cells as an isoform-specific receptor for vascular endothelial growth factor. Cell.

[50] Xu Y., Yuan L., Mak J., Pardanaud L., Caunt M., Kasman I., Larrivée B., Del Toro R., Suchting S., Medvinsky A. (2010). Neuropilin-2 mediates VEGF-C-induced lymphatic sprouting together with VEGFR3. J. Cell Biol..

[51] Pellet-Many C., Frankel P., Evans I.M., Herzog B., Jünemann-Ramírez M., Zachary I.C. (2011). Neuropilin-1 mediates PDGF stimulation of vascular smooth muscle cell migration and signalling via p130Cas. Biochem. J..

[52] Evans I.M., Yamaji M., Britton G., Pellet-Many C., Lockie C., Zachary I.C., Frankel P. (2011). Neuropilin-1 signaling through p130Cas tyrosine phosphorylation is essential for growth factor-dependent migration of glioma and endothelial cells. Mol. Cell Biol..

[53] Snuderl M., Batista A., Kirkpatrick N.D., Ruiz de Almodovar C., Riedemann L., Walsh E.C., Anolik R., Huang Y., Martin J.D., Kamoun W. (2013). Targeting placental growth factor/neuropilin 1 pathway inhibits growth and spread of medulloblastoma. Cell.

[54] Ruffini F., D’Atri S., Lacal P.M. (2013). Neuropilin-1 expression promotes invasiveness of melanoma cells through vascular endothelial growth factor receptor-2-dependent and -independent mechanisms. Int. J. Oncol..

[55] Graziani G., Lacal P.M. (2015). Neuropilin-1 as Therapeutic Target for Malignant Melanoma. Front. Oncol..

[56] Ferrara N. (2009). VEGF-A: A critical regulator of blood vessel growth. Eur. Cytokine Netw..

[57] Nagy J.A., Chang S.H., Dvorak A.M., Dvorak H.F. (2009). Why are tumour blood vessels abnormal and why is it important to know?. Br. J. Cancer.

[58] Ferrara N. (2010). Binding to the extracellular matrix and proteolytic processing: Two key mechanisms regulating vascular endothelial growth factor action. Mol. Biol. Cell.

[59] Jain R.K., Booth M.F. (2003). What brings pericytes to tumor vessels?. J. Clin Invest..

[60] Angelo L.S., Kurzrock R. (2007). Vascular endothelial growth factor and its relationship to inflammatory mediators. Clin. Cancer Res..

[61] Stockmann C., Schadendorf D., Klose R., Helfrich I. (2014). The impact of the immune system on tumor: Angiogenesis and vascular remodeling. Front. Oncol..

[62] Dmitrieva O.S., Shilovskiy I.P., Khaitov M.R., Grivennikov S.I. (2016). Interleukins 1 and 6 as Main Mediators of Inflammation and Cancer. Biochemistry (Mosc).

[63] Aguilar-Cazares D., Chavez-Dominguez R., Carlos-Reyes A., Lopez-Camarillo C., Hernadez de la Cruz O.N., Lopez-Gonzalez J.S. (2019). Contribution of Angiogenesis to Inflammation and Cancer. Front. Oncol..

[64] Kerbel R.S. (2008). Tumor angiogenesis. N. Engl. J. Med..

[65] Ferrara N., Adamis A.P. (2016). Ten years of anti-vascular endothelial growth factor therapy. Nat. Rev. Drug Discov..

[66] Jayson G.C., Kerbel R., Ellis L.M., Harris A.L. (2016). Antiangiogenic therapy in oncology: Current status and future directions. Lancet.

[67] Lacal P.M., Graziani G. (2018). Therapeutic implication of vascular endothelial growth factor receptor-1 (VEGFR-1) targeting in cancer cells and tumor microenvironment by competitive and non-competitive inhibitors. Pharmacol. Res..

[68] Albrecht I., Kopfstein L., Strittmatter K., Schomber T., Falkevall A., Hagberg C.E., Lorentz P., Jeltsch M., Alitalo K., Eriksson U. (2010). Suppressive effects of vascular endothelial growth factor-B on tumor growth in a mouse model of pancreatic neuroendocrine tumorigenesis. PLoS ONE.

[69] Yang X., Zhang Y., Hosaka K., Andersson P., Wang J., Tholander F., Cao Z., Morikawa H., Tegnér J., Yang Y. (2015). VEGF- B promotes cancer metastasis through a VEGF-A-independent mechanism and serves as a marker of poor prognosis for cancer patients. Proc. Natl. Acad. Sci. USA.

[70] Marigo I., Bronte V. (2008). Tumor-induced tolerance and immune suppression by myeloid derived suppressor cells. Immunol. Rev..

[71] Albonici L., Giganti M.G., Modesti A., Manzari V., Bei R. (2019). Multifaceted Role of the Placental Growth Factor (PlGF) in the Antitumor Immune Response and Cancer Progression. Int. J. Mol. Sci..

[72] Lapeyre-Prost A., Terme M., Pernot S., Pointet A.L., Voron T., Tartour E., Taieb J. (2017). Immunomodulatory Activity of VEGF in Cancer. Int. Rev. Cell. Mol. Biol..

[73] Bingle L., Brown N.J., Lewis C.E. (2002). The role of tumour-associated macrophages in tumour progression: Implications for new anticancer therapies. J. Pathol..

[74] Coussens L.M., Werb Z. (2001). Inflammatory cells and cancer think different!. J. Exp. Med..

[75] Mantovani A., Ruco L. (1992). The origin and function of tumor-associated macrophages. Immunol. Today.

[76] Linde N., Lederle W., Depner S., van Rooijen N., Gutschalk C.M., Mueller M.M. (2012). Vascular endothelial growth factor induced skin carcinogenesis depends on recruitment and alternative activation of macrophages. J. Pathol..

[77] Incio J., Tam J., Rahbari N.N., Suboj P., McManus D.T., Chin S.M., Vardam T.D., Batista A., Babykutty S., Jung K. (2016). PlGF/VEGFR-1 Signaling Promotes Macrophage Polarization and Accelerated Tumor Progression in Obesity. Clin. Cancer Res..

[78] He C., Zhu K., Bai X., Li Y., Sun D., Lang Y., Ning J., Sun F., Qu C., Xu S. (2018). Placental Growth Factor Mediates Crosstalk Between Lung Cancer Cells and Tumor-Associated Macrophages in Controlling Cancer Vascularization and Growth. Cell. Physiol Biochem..

[79] Tamura T., Kurishima K., Kagohashi K., Ishikawa H., Satoh H., Hizawa N. (2015). Specific organ metastases and survival in metastatic non-small-cell lung cancer. Mol. Clin. Oncol..

[80] Popper H.H. (2016). Progression and metastasis of lung cancer. Cancer Metastasis Rev..

[81] Frezzetti D., Gallo M., Roma C., D’Alessio A., Maiello M.R., Bevilacqua S., Normanno N., De Luca A. (2016). Vascular Endothelial Growth Factor A Regulates the Secretion of Different Angiogenic Factors in Lung Cancer Cells. J. Cell. Physiol..

[82] Cohen M.H., Gootenberg J., Keegan P., Pazdur R. (2007). FDA drug approval summary: Bevacizumab (Avastin) plus Carboplatin and Paclitaxel as first-line treatment of advanced/metastatic recurrent nonsquamous non-small cell lung cancer. Oncologist.

[83] Zhang S.D., McCrudden C.M., Kwok H.F. (2015). Prognostic significance of combining VEGFA, FLT1 and KDR mRNA expression in lung cancer. Oncol. Lett..

[84] Shimoda L.A., Semenza G.L. (2011). HIF and the lung: Role of hypoxia-inducible factors in pulmonary development and disease. Am. J. Respir. Crit. Care Med..

[85] Qian J., Bai H., Gao Z., Dong Y.U., Pei J., Ma M., Han B. (2016). Downregulation of HIF-1α inhibits the proliferation and invasion of non-small cell lung cancer NCI-H157 cells. Oncol. Lett..

[86] Zhu X., Li H., Long L., Hui L., Chen H., Wang X., Shen H., Xu W. (2012). miR-126 enhances the sensitivity of non-small cell lung cancer cells to anticancer agents by targeting vascular endothelial growth factor A. Acta Biochim. Biophys. Sin. (Shanghai).

[87] Gu A., Lu J., Wang W., Shi C., Han B., Yao M. (2016). Role of miR-497 in VEGF-A-mediated cancer cell growth and invasion in non-small cell lung cancer. Int. J. Biochem. Cell Biol..

[88] Pan J.Y., Sun C.C., Bi Z.Y., Chen Z.L., Li S.J., Li Q.Q., Wang Y.X., Bi Y.Y., Li D.J. (2017). miR-206/133b Cluster: A Weapon against Lung Cancer?. Mol. Ther. Nucleic Acids.

[89] Liu L., Bi N., Wu L., Ding X., Men Y., Zhou W., Li L., Zhang W., Shi S., Song Y. (2017). MicroRNA-29c functions as a tumor suppressor by targeting VEGFA in lung adenocarcinoma. Mol. Cancer.

[90] Zhou Y., Li S., Li J., Wang D., Li Q. (2017). Effect of microRNA-135a on Cell Proliferation, Migration, Invasion, Apoptosis and Tumor Angiogenesis Through the IGF-1/PI3K/Akt Signaling Pathway in Non-Small Cell Lung Cancer. Cell Physiol. Biochem..

[91] Liu H., Chen Y., Li Y., Li C., Qin T., Bai M., Zhang Z., Jia R., Su Y., Wang C. (2019). miR-195 suppresses metastasis and angiogenesis of squamous cell lung cancer by inhibiting the expression of VEGF. Mol. Med. Rep..

[92] Pan B., Shen J., Cao J., Zhou Y., Shang L., Jin S., Cao S., Che D., Liu F., Yu Y. (2015). Interleukin-17 promotes angiogenesis by stimulating VEGF production of cancer cells via the STAT3/GIV signaling pathway in non-small-cell lung cancer. Sci. Rep..

[93] Abou Faycal C., Gazzeri S., Eymin B. (2019). A VEGF-A/SOX2/SRSF2 network controls VEGFR1 pre-mRNA alternative splicing in lung carcinoma cells. Sci. Rep..

[94] Soro S., Orecchia A., Morbidelli L., Lacal P.M., Morea V., Ballmer-Hofer K., Ruffini F., Ziche M., D’Atri S., Zambruno G. (2008). A proangiogenic peptide derived from vascular endothelial growth factor receptor-1 acts through alpha5beta1 integrin. Blood.

[95] Zhang W., Zhang T., Lou Y., Yan B., Cui S., Jiang L., Han B. (2015). Placental growth factor promotes metastases of non-small cell lung cancer through MMP9. Cell. Physiol. Biochem..

[96] Wang Z., Liu T. (2018). Placental growth factor signaling regulates isoform splicing of vascular endothelial growth factor A in the control of lung cancer cell metastasis. Mol. Cell. Biochem..

[97] Nakazawa K., Kurishima K., Tamura T., Kagohashi K., Ishikawa H., Satoh H., Hizawa N. (2012). Specific organ metastases and survival in small cell lung cancer. Oncol. Lett..

[98] Lin H., Li L., Luo S., Zhou S., Shen R., Yang H., Chen H., Xie X. (2017). Efficacy and safety of angiogenesis inhibitors in small-cell lung cancer. Oncotarget.

[99] Robinson B.W., Musk A.W., Lake R.A. (2005). Malignant mesothelioma. Lancet.

[100] Pompeo E., Albonici L., Doldo E., Orlandi A., Manzari V., Modesti A., Mineo T.C. (2009). Placenta growth factor expression has prognostic value in malignant pleural mesothelioma. Ann. Thorac. Surg..

[101] Albonici L., Doldo E., Palumbo C., Orlandi A., Bei R., Pompeo E., Mineo T.C., Modesti A., Manzari V. (2009). Placenta growth factor is a survival factor for human malignant mesothelioma cells. Int. J. Immunopathol. Pharmacol..

[102] Kaplan R.N., Riba R.D., Zacharoulis S., Bramley A.H., Vincent L., Costa C., MacDonald D.D., Jin D.K., Shido K., Kerns S.A. (2005). VEGFR1-positive haematopoietic bone marrow progenitors initiate the pre-metastatic niche. Nature.

[103] Dawson M.R., Duda D.G., Chae S.S., Fukumura D., Jain R.K. (2009). VEGFR1 activity modulates myeloid cell infiltration in growing lung metastases but is not required for spontaneous metastasis formation. PLoS ONE.

[104] Kim J., Sinn D.H., Choi M.S., Kang W., Gwak G.Y., Paik Y.H., Lee J.H., Koh K.C., Paik S.W. (2019). Hepatocellular Carcinoma With Extrahepatic Metastasis: Are There Still Candidates for Transarterial Chemoembolization as an Initial Treatment?. PLoS ONE.

[105] Morse M.A., Sun W., Kim R., He A.R., Abada P.B., Mynderse M., Finn R.S. (2019). The Role of Angiogenesis in Hepatocellular Carcinoma. Clin. Cancer Res..

[106] Poon R.T., Fan S.T., Wong J. (2001). Clinical implications of circulating angiogenic factors in cancer patients. J. Clin. Oncol..

[107] Schoenleber S.J., Kurtz D.M., Talwalkar J.A., Roberts L.R., Gores G.J. (2009). Prognostic role of vascular endothelial growth factor in hepatocellular carcinoma: Systematic review and meta-analysis. Br. J. Cancer.

[108] Llovet J.M., Peña C.E., Lathia C.D., Shan M., Meinhardt G., Bruix J., SHARP Investigators Study Group (2012). Plasma biomarkers as predictors of outcome in patients with advanced hepatocellular carcinoma. Clin. Cancer Res..

[109] Zhan P., Qian Q., Yu L.K. (2013). Serum VEGF level is associated with the outcome of patients with hepatocellular carcinoma: A meta-analysis. Hepatobiliary Surg. Nutr..

[110] Kanda M., Nomoto S., Nishikawa Y., Sugimoto H., Kanazumi N., Takeda S., Nakao A. (2008). Correlations of the expression of vascular endothelial growth factor B and its isoforms in hepatocellular carcinoma with clinico-pathological parameters. J. Surg. Oncol..

[111] Ghosh A., Dasgupta D., Ghosh A., Roychoudhury S., Kumar D., Gorain M., Butti R., Datta S., Agarwal S., Gupta S. (2017). MiRNA199a-3p suppresses tumor growth, migration, invasion and angiogenesis in hepatocellular carcinoma by targeting VEGFA, VEGFR1, VEGFR2, HGF and MMP2. Cell Death Dis..

[112] Van de Veire S., Stalmans I., Heindryckx F., Oura H., Tijeras-Raballand A., Schmidt T., Loges S., Albrecht I., Jonckx B., Vinckier S. (2010). Further pharmacological and genetic evidence for the efficacy of PlGF inhibition in cancer and eye disease. Cell.

[113] Vandewynckel Y.P., Laukens D., Devisscher L., Bogaerts E., Paridaens A., Van den Bussche A., Raevens S., Verhelst X., Van Steenkiste C., Jonckx B. (2016). Placental growth factor inhibition modulates the interplay between hypoxia and unfolded protein response in hepatocellular carcinoma. BMC Cancer.

[114] Heindryckx F., Bogaerts E., Coulon S.H., Devlies H., Geerts A.M., Libbrecht L., Stassen J.M., Carmeliet P., Colle I.O., Van Vlierberghe H.R. (2012). Inhibition of the placental growth factor decreases burden of cholangiocarcinoma and hepatocellular carcinoma in a transgenic mouse model. Eur. J. Gastroenterol. Hepatol..

[115] Yi Z.Y., Feng L.J., Xiang Z., Yao H. (2011). Vascular endothelial growth factor receptor-1 activation mediates epithelial to mesenchymal transition in hepatocellular carcinoma cells. J. Invest. Surg..

[116] Li T., Zhu Y., Han L., Ren W., Liu H., Qin C. (2015). VEGFR-1 activation-induced MMP-9-dependent invasion in hepatocellular carcinoma. Future Oncol..

[117] Ng I.O., Poon R.T., Lee J.M., Fan S.T., Ng M., Tso W.K. (2001). Microvessel density, vascular endothelial growth factor and its receptors Flt-1 and Flk-1/KDR in hepatocellular carcinoma. Am. J. Clin. Pathol..

[118] Li T., Zhu Y., Qin C.Y., Yang Z., Fang A., Xu S., Ren W. (2012). Expression and prognostic significance of vascular endothelial growth factor receptor 1 in hepatocellular carcinoma. J. Clin. Pathol..

[119] Simone V., Brunetti O., Lupo L., Testini M., Maiorano E., Simone M., Longo V., Rolfo C., Peeters M., Scarpa A. (2017). Targeting Angiogenesis in Biliary Tract Cancers: An Open Option. Int. J. Mol. Sci..

[120] Zhang J., Han C., Zhu H., Song K., Wu T. (2013). miR-101 inhibits cholangiocarcinoma angiogenesis through targeting vascular endothelial growth factor (VEGF). Am. J. Pathol..

[121] Xu D., Li J., Jiang F., Cai K., Ren G. (2019). The Effect and Mechanism of Vascular Endothelial Growth Factor (VEGF) on Tumor Angiogenesis in Gallbladder Carcinoma. Iran. J. Public Health.

[122] Letelier P., Garcia P., Leal P., Ili C., Buchegger K., Riquelme I., Sandoval A., Tapia O., Roa J.C. (2014). Immunohistochemical expression of vascular endothelial growth factor A in advanced gallbladder carcinoma. Appl. Immunohistochem. Mol. Morphol..

[123] Li H., Jin Y., Hu Y., Jiang L., Liu F., Zhang Y., Hao Y., Chen S., Wu X., Liu Y. (2018). The PLGF/c-MYC/miR-19a axis promotes metastasis and stemness in gallbladder cancer. Cancer Sci..

[124] Lee Y.J., Karl D.L., Maduekwe U.N., Rothrock C., Ryeom S., D’Amore P.A., Yoon S.S. (2010). Differential effects of VEGFR-1 and VEGFR-2 inhibition on tumor metastases based on host organ environment. Cancer Res..

[125] Lee J.W., Stone M.L., Porrett P.M., Thomas S.K., Komar C.A., Li J.H., Delman D., Graham K., Gladney W.L., Hua X. (2019). Hepatocytes direct the formation of a pro-metastatic niche in the liver. Nature.

[126] Escudier B., Porta C., Schmidinger M., Rioux-Leclercq N., Bex A., Khoo V., Grünwald V., Gillessen S., Horwich A., ESMO Guidelines Committee (2019). Electronic address: Clinicalguidelines@esmo.org. Renal cell carcinoma: ESMO Clinical Practice Guidelines for diagnosis, treatment and follow-up. Ann. Oncol..

[127] Choueiri T.K., Motzer R.J. (2017). Systemic Therapy for Metastatic Renal-Cell Carcinoma. N. Engl. J. Med..

[128] Ivan M., Kondo K., Yang H., Kim W., Valiando J., Ohh M., Salic A., Asara J.M., Lane W.S., Kaelin W.G. (2001). HIFalpha targeted for VHL-mediated destruction by proline hydroxylation: Implications for O_2_ sensing. Science.

[129] Jaakkola P., Mole D.R., Tian Y.M., Wilson M.I., Gielbert J., Gaskell S.J., von Kriegsheim A., Hebestreit H.F., Mukherji M., Schofield C.J. (2001). Targeting of HIF-alpha to the von Hippel-Lindau ubiquitylation complex by O2-regulated prolyl hydroxylation. Science.

[130] Xian W., Zheng H., Wu W.J. (2015). Predictive value of vascular endothelial growth factor polymorphisms on the risk of renal cell carcinomas. Genet. Mol Res..

[131] Kawai Y., Sakano S., Korenaga Y., Eguchi S., Naito K. (2007). Associations of Single Nucleotide Polymorphisms in the Vascular Endothelial Growth Factor Gene with the Characteristics and Prognosis of Renal Cell Carcinomas. Eur Urol..

[132] Zhong W., Wang X., Pan B., Su Z. (2014). Association of vascular endothelial growth factor polymorphisms with clinical outcome of renal cell carcinoma patients. Tumour Biol..

[133] Gong M., Dong W., Shi Z., Qiu S., Yuan R. (2017). Vascular endothelial growth factor gene polymorphisms and the risk of renal cell carcinoma: Evidence from eight case-control studies. Oncotarget.

[134] Tang J., Qin Z., Li X., Han P., Wang F., Yang C., Li R., Wang K., Tang M., Wang W. (2017). Association between vascular endothelial growth factor gene polymorphisms and the risk and prognosis of renal cell carcinoma: A systematic review and meta-analysis. Oncotarget.

[135] Chen Y.S., Meng F., Li H.L., Liu Q.H., Hou P.F., Bai J., Zheng J.N. (2016). Dicer suppresses MMP-2-mediated invasion and VEGFA-induced angiogenesis and serves as a promising prognostic biomarker in human clear cell renal cell carcinoma. Oncotarget.

[136] Rivet J., Mourah S., Murata H., Mounier N., Pisonero H., Mongiat-Artus P., Teillac P., Calvo F., Janin A., Dosquet C. (2008). VEGF and VEGFR-1 are coexpressed by epithelial and stromal cells of renal cell carcinoma. Cancer.

[137] Gunningham S.P., Currie M.J., Han C., Turner K., Scott P.A., Robinson B.A., Harris A.L., Fox S.B. (2001). Vascular endothelial growth factor-B and vascular endothelial growth factor-C expression in renal cell carcinomas: Regulation by the von Hippel-Lindau gene and hypoxia. Cancer Res..

[138] Kusmartsev S., Eruslanov E., Kübler H., Tseng T., Sakai Y., Su Z., Kaliberov S., Heiser A., Rosser C., Dahm P. (2008). Oxidative stress regulates expression of VEGFR1 in myeloid cells: Link to tumor-induced immune suppression in renal cell carcinoma. J. Immunol..

[139] Motzer R.J., Michaelson M.D., Redman B.G., Hudes G.R., Wilding G., Figlin R.A., Ginsberg M.S., Kim S.T., Baum C.M., DePrimo S.E. (2006). Activity of SU11248, a multitargeted inhibitor of vascular endothelial growth factor receptor and platelet-derived growth factor receptor, in patients with metastatic renal cell carcinoma. J. Clin Oncol.

[140] Bessho H., Wong B., Huang D., Siew E.Y., Huang D., Tan J., Ong C.K., Tan S.Y., Matsumoto K., Iwamura M. Inhibition of Placental Growth Factor in Renal Cell Carcinoma Anticancer Res. 2015, 35, 531–542. 35.

[141] Taylor O.G., Brzozowski J.S., Skelding K.A. (2019). Glioblastoma Multiforme: An Overview of Emerging Therapeutic Targets. Front. Oncol..

[142] Davis M.E. (2016). Glioblastoma: Overview of Disease and Treatment. Clin. J. Oncol. Nurs..

[143] Sun Q., Xu R., Xu H., Wang G., Shen X., Jiang H. (2017). Extracranial metastases of high-grade glioma: The clinical characteristics and mechanism. World, J. Surg. Oncol..

[144] Baumgarten P., Blank A.E., Franz K., Hattingen E., Dunst M., Zeiner P., Hoffmann K., Bähr O., Mäder L., Goeppert B. (2016). Differential expression of vascular endothelial growth factor A, its receptors VEGFR-1, -2, and -3 and co-receptors neuropilin-1 and -2 does not predict bevacizumab response in human astrocytomas. Neuro Oncol..

[145] Atzori M.G., Tentori L., Ruffini F., Ceci C., Lisi L., Bonanno E., Scimeca M., Eskilsson E., Daubon T., Miletic H. (2017). The anti-vascular endothelial growth factor receptor-1 monoclonal antibody D16F7 inhibits invasiveness of human glioblastoma and glioblastoma stem cells. J. Exp. Clin. Cancer Res..

[146] D’Alessio A., Proietti G., Lama G., Biamonte F., Lauriola L., Moscato U., Vescovi A., Mangiola A., Angelucci C., Sica G. (2016). Analysis of angiogenesis related factors in glioblastoma, peritumoral tissue and their derived cancer stem cells. Oncotarget.

[147] Jiang K., Wang Y.P., Wang X.D., Hui X.B., Ding L.S., Liu J., Liu D. (2017). Fms related tyrosine kinase 1 (Flt1) functions as an oncogene and regulates glioblastoma cell metastasis by regulating sonic hedgehog signaling. Am. J. Cancer Res..

[148] Atzori M.G., Tentori L., Ruffini F., Ceci C., Bonanno E., Scimeca M., Lacal P.M., Graziani G. (2018). The Anti-Vascular Endothelial Growth Factor Receptor-1 Monoclonal Antibody D16F7 Inhibits Glioma Growth and Angiogenesis In Vivo. J. Pharmacol. Exp. Ther..

[149] Dello Russo C., Lisi L., Tentori L., Navarra P., Graziani G., Combs C.K. (2017). Exploiting Microglial Functions for the Treatment of Glioblastoma. Curr. Cancer Drug Targets.

[150] Turkowski K., Brandenburg S., Mueller A., Kremenetskaia I., Bungert A.D., Blank A., Felsenstein M., Vajkoczy P. (2018). VEGF as a modulator of the innate immune response in glioblastoma. Glia.

[151] Piao Y., Liang J., Holmes L., Zurita A.J., Henry V., Heymach J.V., de Groot J.F. (2012). Glioblastoma resistance to anti-VEGF therapy is associated with myeloid cell infiltration, stem cell accumulation, and a mesenchymal phenotype. Neuro Oncol..

[152] Gabrusiewicz K., Liu D., Cortes-Santiago N., Hossain M.B., Conrad C.A., Aldape K.D., Fuller G.N., Marini F.C., Alonso M., Idoate M.A. (2014). Anti-vascular endothelial growth factor therapy-induced glioma invasion is associated with accumulation of Tie2-expressing monocytes. Oncotarget.

[153] Lu-Emerson C., Snuderl M., Kirkpatrick N.D., Goveia J., Davidson C., Huang Y., Riedemann L., Taylor J., Ivy P., Duda D.G. (2013). Increase in tumor-associated macrophages after antiangiogenic therapy is associated with poor survival among patients with recurrent glioblastoma. Neuro Oncol..

[154] Lisi L., Ciotti G.M.P., Chiavari M., Ruffini F., Lacal P.M., Graziani G., Navarra P. (2020). Vascular endothelial growth factor receptor 1 in glioblastoma-associated microglia/macrophages. Oncol. Rep..

[155] Keung E.Z., Gershenwald J.E. (2018). The eighth edition American Joint Committee on Cancer (AJCC) melanoma staging system: Implications for melanoma treatment and care. Expert Rev. Anticancer Ther..

[156] Enomoto L.M., Levine E.A., Shen P., Votanopoulos K.I. (2020). Role of Surgery for Metastatic Melanoma. Surg. Clin. North. Am..

[157] Lacal P.M., Failla C.M., Pagani E., Odorisio T., Schietroma C., Falcinelli S., Zambruno G., D’Atri S. (2000). Human melanoma cells secrete and respond to placenta growth factor and vascular endothelial growth factor. J. Invest. Dermatol..

[158] Frank N.Y., Schatton T., Kim S., Zhan Q., Wilson B.J., Ma J., Saab K.R., Osherov V., Widlund H.R., Gasser M. (2011). VEGFR-1 expressed by malignant melanoma-initiating cells is required for tumor growth. Cancer Res..

[159] Lacal P.M., Ruffini F., Pagani E., D’Atri S. (2005). An autocrine loop directed by the vascular endothelial growth factor promotes invasiveness of human melanoma cells. Int. J. Oncol..

[160] Graziani G., Ruffini F., Tentori L., Scimeca M., Dorio A.S., Atzori M.G., Failla C.M., Morea V., Bonanno E., D’Atri S. (2016). Antitumor activity of a novel anti-vascular endothelial growth factor receptor-1 monoclonal antibody that does not interfere with ligand binding. Oncotarget.

[161] Atzori M.G., Ceci C., Ruffini F., Trapani M., Barbaccia M.L., Tentori L., D’Atri S., Lacal P.M., Graziani G. (2020). Role of VEGFR-1 in melanoma acquired resistance to the BRAF inhibitor vemurafenib. J. Cell. Mol. Med..

[162] Marcellini M., De Luca N., Riccioni T., Ciucci A., Orecchia A., Lacal P.M., Ruffini F., Pesce M., Cianfarani F., Zambruno G. (2006). Increased melanoma growth and metastasis spreading in mice overexpressing placenta growth factor. Am. J. Pathol..

[163] Levati L., Ruffini F., Muzi A., Umezawa K., Graziani G., D’Atri S., Lacal P.M. (2011). Placenta growth factor induces melanoma resistance to temozolomide through a mechanism that involves the activation of the transcription factor NF-κB. Int. J. Oncol..

[164] Evola F.R., Costarella L., Pavone V., Caff G., Cannavò L., Sessa A., Avondo S., Sessa G. (2017). Biomarkers of Osteosarcoma, Chondrosarcoma, and Ewing Sarcoma. Front. Pharmacol..

[165] Meazza C., Scanagatta P. (2016). Metastatic osteosarcoma: A challenging multidisciplinary treatment. Expert Rev. Anticancer Ther..

[166] Luetke A., Meyers P.A., Lewis I., Juergens H. (2014). Osteosarcoma treatment—where do we stand? A state of the art review. Cancer Treat. Rev..

[167] Allen-Rhoades W., Whittle S.B., Rainusso N. (2018). Pediatric Solid Tumors in Children and Adolescents: An Overview. Pediatr. Rev..

[168] Whelan J.S., Davis L.E. (2018). Osteosarcoma, Chondrosarcoma, and Chordoma. J. Clin. Oncol..

[169] Worch J., Ranft A., DuBois S.G., Paulussen M., Juergens H., Dirksen U. (2018). Age dependency of primary tumor sites and metastases in patients with Ewing sarcoma. Pediatr. Blood Cancer.

[170] Yang J., Yang D., Sun Y., Sun B., Wang G., Trent J.C., Araujo D.M., Chen K., Zhang W. (2011). Genetic amplification of the vascular endothelial growth factor (VEGF) pathway genes, including VEGFA, in human osteosarcoma. Cancer.

[171] Rastogi S., Kumar R., Sankineani S.R., Marimuthu K., Rijal L., Prakash S., Jalan D., Khan S.A., Sharma M.C. (2012). Role of vascular endothelial growth factor as a tumour marker in osteosarcoma: A prospective study. Intl. Orthop..

[172] Chen D., Zhang Y.J., Zhu K.W., Wang W.C. (2013). A systematic review of vascular endothelial growth factor expression as a biomarker of prognosis in patients with osteosarcoma. Tumour Biol..

[173] Hu Y.Y., Du X.Y., Zhan A.L., Zhou L., Jiang Q., Niu Y.M., Shen M. (2016). Vascular endothelial growth factor polymorphisms are associated with osteosarcoma susceptibility. Oncotarget.

[174] Zhao H., Wu Y., Chen Y., Liu H. (2015). Clinical significance of hypoxia-inducible factor 1 and VEGF-A in osteosarcoma. Int J. Clin Oncol..

[175] Ohba T., Cates J.M., Cole H.A., Slosky D.A., Haro H., Ando T., Schwartz H.S., Schoenecker J.G. (2014). Autocrine VEGF/VEGFR1 signaling in a subpopulation of cells associates with aggressive osteosarcoma. Mol Cancer Res..

[176] Sarver A.L., Thayanithy V., Scott M.C., Cleton-Jansen A.M., Hogendoorn P.C., Modiano J.F., Subramanian S. (2013). MicroRNAs at the human 14q32 locus have prognostic significance in osteosarcoma. Orphanet J. Rare Dis..

[177] Zhang L., Lv Z., Xu J., Chen C., Ge Q., Li P., Wei D., Wu Z., Sun X. (2018). MicroRNA-134 inhibits osteosarcoma angiogenesis and proliferation by targeting the VEGFA/VEGFR1 pathway. FEBS J..

[178] Niu J., Sun Y., Guo Q., Niu D., Liu B. (2016). miR-1 Inhibits Cell Growth, Migration, and Invasion by Targeting VEGFA in Osteosarcoma Cells. Dis Markers.

[179] Guan H., Zhou Z., Cao Y., Duan X., Kleinerman E.S. (2009). VEGF165 promotes the osteolytic bone destruction of ewing’s sarcoma tumors by upregulating RANKL. Oncol Res..

[180] Huang G., Zhou Z., Wang H., Kleinerman E.S. (2012). CAPER-α alternative splicing regulates the expression of vascular endothelial growth factor₁₆₅ in Ewing sarcoma cells. Cancer.

[181] Fujiwara T., Fukushi J., Yamamoto S., Matsumoto Y., Setsu N., Oda Y., Yamada H., Okada S., Watari K., Ono M. (2011). Macrophage infiltration predicts a poor prognosis for human ewing sarcoma. Am. J. Pathol..

[182] Lee H.P., Lin C.Y., Shih J.S., Fong Y.C., Wang S.W., Li T.M., Tang C.H. (2015). Adiponectin promotes VEGF-A-dependent angiogenesis in human chondrosarcoma through PI3K, Akt, mTOR, and HIF-α pathway. Oncotarget.

[183] Lin C.Y., Tzeng H.E., Li T.M., Chen H.T., Lee Y., Yang Y.C., Wang S.W., Yang W.H., Tang C.H. (2017). WISP-3 inhibition of miR-452 promotes VEGF-A expression in chondrosarcoma cells and induces endothelial progenitor cells angiogenesis. Oncotarget.

[184] Ducreux M., Cuhna A.S., Caramella C., Hollebecque A., Burtin P., Goéré D., Seufferlein T., Haustermans K., Van Laethem J.L., Conroy T. (2015). Cancer of the pancreas: ESMO Clinical Practice Guidelines for diagnosis, treatment and follow-up. Ann. Oncol..

[185] Deeb A., Haque S.U., Olowokure O. (2015). Pulmonary metastases in pancreatic cancer, is there a survival influence?. J. Gastrointest. Oncol..

[186] Peixoto R.D., Speers C., McGahan C.E., Renouf D.J., Schaeffer D.F., Kennecke H.F. (2015). Prognostic factors and sites of metastasis in unresectable locally advanced pancreatic cancer. Cancer Med..

[187] Seo Y., Baba H., Fukuda T., Takashima M., Sugimachi K. (2000). High expression of vascular endothelial growth factor is associated with liver metastasis and a poor prognosis for patients with ductal pancreatic adenocarcinoma. Cancer.

[188] Von Marschall Z., Cramer T., Höcker M., Burde R., Plath T., Schirner, M., Heidenreich R., Breier G., Riecken E.O., Wiedenmann B. (2000). De novo expression of vascular endothelial growth factor in human pancreatic cancer: Evidence for an autocrine mitogenic loop. Gastroenterology.

[189] Shi S., Xu J., Zhang B., Ji S., Xu W., Liu J., Jin K., Liang D., Liang C., Liu L. (2016). VEGF Promotes Glycolysis in Pancreatic Cancer via HIF1α Up-Regulation. Curr. Mo.l Med..

[190] Costache M.I., Iordache S., Costache C.A., Dragos E., Dragos A., Saftoiu A. (2017). Molecular Analysis of Vascular Endothelial Growth Factor (VEGF) Receptors in EUS-guided Samples Obtained from Patients with Pancreatic Adenocarcinoma. J. Gastrointestin. Liver Dis..

[191] Huang J., Mei H., Tang Z., Li J., Zhang X., Lu Y., Huang F., Jin Q., Wang Z. (2017). Triple-amiRNA VEGFRs inhibition in pancreatic cancer improves the efficacy of chemotherapy through EMT regulation. J. Control. Release.

[192] D’souza S., Addepalli V. (2018). Preventive measures in oral cancer: An overview. Biomed. Pharmacother..

[193] Irani S. (2016). Distant metastasis from oral cancer: A review and molecular biologic aspects. J. Int. Soc. Prev. Community Dent..

[194] Zhao S.F., Yang X.D., Lu M.X., Sun G.W., Wang Y.X., Zhang Y.K., Pu Y.M., Tang E.Y. (2013). Prognostic significance of VEGF immunohistochemical expression in oral cancer: A meta-analysis of the literature. Tumor Biol..

[195] Lin Y.W., Huang S.T., Wu J.C., Chu T.H., Huang S.C., Lee C.C., Tai M.H. (2019). HDGF/HIF-1α/VEGF axis in oral cancer impacts disease prognosis. BMC Cancer.

[196] Costache M.I., Ioan M., Iordache S., Ene D., Costache C.A., Saftoiu A. (2015). VEGF Expression in Pancreatic Cancer and Other Malignancies: A Review of the Literature. Rom. J. Intern. Med..

[197] Subarnbhesaj A., Miyauchi M., Chanbora C., Mikuriya A., Nguyen P.T., Furusho H., Ayuningtyas N.F., Fujita M., Toratani S., Takechi M. (2017). Roles of VEGF-Flt-1 signaling in malignant behaviors of oral squamous cell carcinoma. PLoS ONE.

[198] Tiasto V., Mikhailova V., Gulaia V., Vikhareva V., Zorin B., Kalitnik A., Kagansky A. (2018). Esophageal cancer research today and tomorrow: Lesson from algae and other perspectives. AIMS Genet..

[199] Huang T.X., Fu L. (2019). The immune landscape of esophageal cancer. Cancer Commun. (Lond).

[200] Hoeppner J., Kulemann B. (2017). Circulating Tumor Cells in Esophageal Cancer. Oncol. Res. Treat..

[201] Kilic E., Schild S.E., Thorns C., Bajrovic A., Rades D. (2014). Prognostic role of vascular endothelial growth factor and its receptor-1 in patients with esophageal cancer. Anticancer Res..

[202] Yang P.W., Hsieh M.S., Huang Y.C., Hsieh C.Y., Chiang T.H., Lee J.M. (2014). Genetic variants of EGF and VEGF predict prognosis of patients with advanced esophageal squamous cell carcinoma. PLoS ONE.

[203] Song Z., Wu Y., Yang J., Yang D., Fang X. (2017). Progress in the treatment of advanced gastric cancer. Tumour Biol..

[204] Shimizu D., Kanda M., Kodera Y. (2018). Emerging evidence of the molecular landscape specific for hematogenous metastasis from gastric cancer. World J. Gastrointest. Oncol..

[205] Mimori K., Fukagawa T., Kosaka Y., Kita Y., Ishikawa K., Etoh T., Iinuma H., Sasako M., Mori M. (2008). Hematogenous metastasis in gastric cancer requires isolated tumor cells and expression of vascular endothelial growth factor receptor-1. Clin. Cancer Res..

[206] Li T., Yu J., Luo X., Ren W., Zhang Y., Cao B. (2018). VEGFR-2 as a novel predictor of survival in gastric cancer: A systematic review and meta-analysis. Pathol. Res. Pract..

[207] Chen S., Zhang X., Peng J., Zhai E., He Y., Wu H., Chen C., Ma J., Wang Z., Cai S. (2016). VEGF promotes gastric cancer development by upregulating CRMP4. Oncotarget.

[208] Ding S., Lin S., Dong X., Yang X., Qu H., Huang S., Liu W., Zhou L., Liu D. (2005). Potential Prognostic Value of Circulating Levels of Vascular Endothelial Growth factor-A in Patients with Gastric Cancer. In Vivo.

[209] Akrami H., Mahmoodi F., Havasi S., Sharifi A. (2016). PlGF knockdown inhibited tumor survival and migration in gastric cancer cell via PI3K/Akt and p38MAPK pathways. Cell. Biochem. Funct..

[210] Mármol I., Sánchez-de-Diego C., Pradilla Dieste A., Cerrada E., Rodriguez Yoldi M.J. (2017). Colorectal Carcinoma: A General Overview and Future Perspectives in Colorectal Cancer. Int. J. Mol. Sci..

[211] Pretzsch E., Bösch F., Neumann J., Ganschow P., Bazhin A., Guba M., Werner J., Angele M. (2019). Mechanisms of Metastasis in Colorectal Cancer and Metastatic Organotropism: Hematogenous versus Peritoneal Spread. J. Oncol..

[212] Fan F., Wey J.S., McCarty M.F., Belcheva A., Liu W., Bauer T.W., Somcio R.J., Wu Y., Hooper A., Hicklin D.J. (2005). Expression and function of vascular endothelial growth factor receptor-1 on human colorectal cancer cells. Oncogene.

[213] Lesslie D.P., Summy J.M., Parikh N.U., Fan F., Trevino J.G., Sawyer T.K., Metcalf C.A., Shakespeare W.C., Hicklin D.J., Ellis L.M. (2006). Vascular Endothelial Growth Factor receptor-1 Mediates Migration of Human Colorectal Carcinoma Cells by Activation of Src Family Kinases. Br. J. Cancer.

[214] Wei S.C., Tsao P.N., Weng M.T., Cao Z., Wong J.M. (2013). Flt-1 in Colorectal Cancer Cells Is Required for the Tumor Invasive Effect of Placental Growth Factor Through a p38-MMP9 Pathway. J. Biomed. Sci..

[215] Cicatiello V., Apicella I., Tudisco L., Tarallo V., Formisano L., Sandomenico A., Kim Y., Bastos-Carvalho A., Orlandi A., Ambati J. (2015). Powerful anti-tumor and anti-angiogenic activity of a new anti-vascular endothelial growth factor receptor 1 peptide in colorectal cancer models. Oncotarget.

[216] Sung C.Y., Son M.W., Ahn T.S., Jung D.J., Lee M.S., Baek M.J. (2012). Expression of placenta growth factor in colorectal carcinomas. J. Korean Soc. Coloproctol..

[217] Jayasinghe C., Simiantonaki N., Kirkpatrick C.J. (2013). VEGF-B expression in colorectal carcinomas and its relevance for tumor progression. Histol. Histopathol..

[218] Bhattacharya R., Ye X.C., Wang R., Ling X., McManus M., Fan F., Boulbes D., Ellis L.M. (2016). Intracrine VEGF Signaling Mediates the Activity of Prosurvival Pathways in Human Colorectal Cancer Cells. Cancer Res..

[219] Bhattacharya R., Fan F., Wang R., Ye X., Xia L., Boulbes D., Ellis L.M. (2017). Intracrine VEGF signalling mediates colorectal cancer cell migration and invasion. Br. J. Cancer..

[220] Freire Valls A., Knipper K., Giannakouri E., Sarachaga V., Hinterkopf S., Wuehrl M., Shen Y., Radhakrishnan P., Klose J., Ulrich A. (2019). VEGFR1_+_ Metastasis-Associated Macrophages Contribute to Metastatic Angiogenesis and Influence Colorectal Cancer Patient Outcome. Clin. Cancer Res..

[221] Ferlay J., Soerjomataram I., Dikshit R., Eser S., Mathers C., Rebelo M., Parkin D.M., Forman D., Bray F. (2015). Cancer incidence and mortality worldwide: Sources, methods and major patterns in GLOBOCAN 2012. Int. J. Cancer.

[222] Cunnick G.H., Jiang W.G., Douglas-Jones T., Watkins G., Gomez K.F., Morgan M.J., Subramanian A., Mokbel K., Mansel R.E. (2008). Lymphangiogenesis and lymph node metastasis in breast cancer. Mol. Cancer.

[223] Aceto N., Bardia A., Miyamoto D.T., Donaldson M.C., Wittner B.S., Spencer J.A., Yu M., Pely A., Engstrom A., Zhu H. (2014). Circulating tumor cell clusters are oligoclonal precursors of breast cancer metastasis. Cell.

[224] Agollah G.D., Wu G., Sevick-Muraca E.M., Kwon S. (2014). In vivo lymphatic imaging of a human inflammatory breast cancer model. J. Cancer.

[225] Jiang M., Qin C., Han M. (2016). Primary Breast Cancer Induces Pulmonary Vascular Hyperpermeability and Promotes Metastasis Via the VEGF–PKC Pathway. Mol. Carcin..

[226] Insa A., Lluch A., Prosper F., Marugan I., Martinez-Agullo A., Garcia-Conde J. (1999). Prognostic factors predicting survival from first recurrence in patients with metastatic breast cancer: Analysis of 439 patients. Breast Cancer Res. Treat..

[227] Chen X., Zheng Z., Chen L., Zheng H. (2017). MAPK, NFκB, and VEGF signaling pathways regulate breast cancer liver metastasis. Oncotarget.

[228] Alhasan L. (2018). MiR-126 Modulates Angiogenesis in Breast Cancer by Targeting VEGF-A –mRNA. Asian Pac. J. Cancer Prev..

[229] Hunter S., Nault B., Chukwunonso Ugwuagbo K., Maiti S., Majumder M. (2019). Mir526b and Mir655 Promote Tumour Associated Angiogenesis and Lymphangiogenesis in Breast Cancer. Cancers.

[230] Chen D.B., Feng L., Hodges J.K., Lechuga T.J., Zhang H. (2017). Human trophoblast-derived hydrogen sulfide stimulates placental artery endothelial cell angiogenesis. Biol. Reprod..

[231] Sbodio J.I., Snyder S.H., Paul B.D. (2019). Regulators of the transsulfuration pathway. Br. J. Pharmacol..

[232] Wang L., Shi H., Liu Y., Zhang W., Duan X., Li M., Shi X., Wang T. (2019). Cystathionine- γ-lyase promotes the metastasis of breast cancer via the VEGF signaling pathway. Int. J. Oncol..

[233] Taylor A.P., Goldenberg D.M. (2007). Role of placenta growth factor in malignancy and evidence that an antagonistic PlGF/Flt-1 peptide inhibits the growth and metastasis of human breast cancer xenografts. Mol. Cancer Ther..

[234] Taylor A.P., Leon E., Goldenberg D.M. (2010). Placental growth factor (PlGF) enhances breast cancer cell motility by mobilising ERK1/2 phosphorylation and cytoskeletal rearrangement. Br. J. Cancer.

[235] Laurent J., Hull E.F., Touvrey C., Kuonen F., Lan Q., Lorusso G., Doucey M.A., Ciarloni L., Imaizumi N., Alghisi G.C. (2011). Proangiogenic factor PlGF programs CD11b(+) myelomonocytes in breast cancer during differentiation of their hematopoietic progenitors. Cancer Res..

[236] Chan D.S., Vieira A.R., Aune D., Bandera E.V., Greenwood D.C., McTiernan A., Navarro Rosenblatt D., Thune I., Vieira R., Norat T. (2014). Body mass index and survival in women with breast cancer-systematic literature review and meta-analysis of 82 follow-up studies. Ann. Oncol..

[237] Sun N., Zhang Q., Xu C., Zhao Q., Ma Y., Lu X., Wang L., Li W. (2014). Molecular regulation of ovarian cancer cell invasion. Tumour Biol..

[238] Lengyel E. (2010). Ovarian cancer development and metastasis. Am. J. Pathol..

[239] Komatsu H., Oishi T., Itamochi H., Shimada M., Sato S., Chikumi J., Sato S., Nonaka M., Sawada M., Wakahara M. (2017). Serum Vascular Endothelial Growth Factor-A as a Prognostic Biomarker for Epithelial Ovarian Cancer. Int. J. Gynecol. Cancer.

[240] Gu F., Li X., Kong J., Pan B., Sun M., Zheng L., Yao Y. (2013). VEGF111b, a new member of VEGFxxxb isoforms and induced by mitomycin C, inhibits angiogenesis. Biochem. Biophys. Res. Commun..

[241] Li X., Gu F., Niu C., Wang Y., Liu Z., Li N., Pan B., He D., Kong J., Zhang S. (2015). VEGF111b, a C-terminal splice variant of VEGF-A and induced by mitomycin C, inhibits ovarian cancer growth. J. Transl. Med..

[242] Horikawa N., Abiko K., Matsumura N., Hamanishi J., Baba T., Yamaguchi K., Yoshioka Y., Koshiyama M., Konishi I. (2017). Expression of Vascular Endothelial Growth Factor in Ovarian Cancer Inhibits Tumor Immunity through the Accumulation of Myeloid-Derived Suppressor Cells. Clin. Cancer Res..

[243] Song N., Liu H., Ma X., Zhang S. (2015). Placental growth factor promotes metastases of ovarian cancer through MiR-543-regulated MMP7. Cell Physiol. Biochem..

[244] Fardi M., Alivand M., Baradaran B., Farshdousti Hagh M., Solali S. (2019). The crucial role of ZEB2: From development to epithelial-to-mesenchymal transition and cancer complexity. J. Cell Physiol..

[245] Song N., Liu H., Ma X., Zhang S. (2016). Placental Growth Factor Promotes Ovarian Cancer Cell Invasion via ZEB2. Cell Physiol. Biochem..

[246] Miyake T., Kumasawa K., Sato N., Takiuchi T., Nakamura H., Kimura T. (2016). Soluble VEGF receptor 1 (sFLT1) induces non-apoptotic death in ovarian and colorectal cancer cells. Sci. Rep..

[247] Haoran L., Xiaohua W., Xi C. (2016). Advances in diagnosis and treatment of metastatic cervical cancer. J. Gynecol. Oncol..

[248] Dang Y.Z., Zhang Y., Li J.P., Hu J., Li W.W., Li P., Wei L.C., Shi M. (2017). High VEGFR1/2 expression levels are predictors of poor survival in patients with cervical cancer. Medicine (Baltimore).

[249] Sawada M., Oishi T., Komatsu H., Sato S., Chikumi J., Nonaka M., Kudoh A., Osaku D., Harada T. (2019). Serum vascular endothelial growth factor A and vascular endothelial growth factor receptor 2 as prognostic biomarkers for uterine cervical cancer. Int. J. Clin. Oncol..

[250] Yoshida K., Suzuki S., Sakata J., Utsumi F., Niimi K., Yoshikawa N., Nishino K., Shibata K., Kikkawa F., Kajiyama H. (2018). The upregulated expression of vascular endothelial growth factor in surgically treated patients with recurrent / radioresistant cervical cancer of the uterus. Oncol. Lett..

[251] Pinheiro C., Garcia E.A., Morais-Santos F., Moreira M.A., Almeida F.M., Jubé L.F., Queiroz G.S., Paula É.C., Andreoli M.A., Villa L.L. (2015). Reprogramming energy metabolism and inducing angiogenesis: Co-expression of monocarboxylate transporters with VEGF family members in cervical adenocarcinomas. BMC Cancer.

[252] Huang W., Zhu S., Liu Q., Li C., Li L. (2014). Placenta growth factor promotes migration through regulating epithelial-mesenchymal transition-related protein expression in cervical cancer. Int. J. Clin. Exp. Pathol..

[253] Wang Q., Steger A., Mahner S., Jeschke U., Heidegger H. (2019). The Formation and Therapeutic Update of Tumor-Associated Macrophages in Cervical Cancer. Int. J. Mol. Sci..

[254] Pedraza-Brindis E.J., Sánchez-Reyes K., Hernández-Flores G., Bravo-Cuellar A., Jave-Suárez L.F., Aguilar-Lemarroy A., Gómez-Lomelí P., López-López B.A., Ortiz-Lazareno P.C. (2016). Culture supernatants of cervical cancer cells induce an M2 phenotypic profile in THP-1 macrophages. Cell Immunol..

[255] De Brot S., Ntekim A., Cardenas R., James V., Allegrucci C., Heery D.M., Bates D.O., Ødum N., Persson J.L., Mongan N.P. (2015). Regulation of vascular endothelial growth factor in prostate cancer. Endocr. Relat. Cancer.

[256] Huang S., Tang Y., Peng X., Cai X., Wa Q., Ren D., Li Q., Luo J., Li L., Zou X. (2016). Acidic extracellular pH promotes prostate cancer bone metastasis by enhancing PC-3 stem cell characteristics, cell invasiveness and VEGF-induced vasculogenesis of BM-EPCs. Oncol. Rep..

[257] Lee C., Whang Y.M., Campbell P., Mulcrone P.L., Elefteriou F., Cho S.W., Park S.I. (2018). Dual targeting c-met and VEGFR2 in osteoblasts suppresses growth and osteolysis of prostate cancer bone metastasis. Cancer Lett..

[258] Bender R.J., Mac Gabhann F. (2015). Dysregulation of the vascular endothelial growth factor and semaphorin ligand-receptor families in prostate cancer metastasis. BMC Syst. Biol..

[259] Grivas N., Goussia A., Stefanou D., Giannakis D. (2016). Microvascular density and immunohistochemical expression of VEGF, VEGFR-1 and VEGFR-2 in benign prostatic hyperplasia, high-grade prostate intraepithelial neoplasia and prostate cancer. Cent. European J. Urol..

[260] Chen Q., Zhao X., Zhang H., Yuan H., Zhu M., Sun Q., Lai X., Wang Y., Huang J., Yan J. (2015). MiR-130b suppresses prostate cancer metastasis through down-regulation of MMP2. Mol. Carcinog..

[261] Mu H.Q., He Y.H., Wang S.B., Yang S., Wang Y.J., Nan C.J., Bao Y.F., Xie Q.P., Chen Y.H. (2019). MiR-130b/TNF-α/NF-κB/VEGFA loop inhibits prostate cancer angiogenesis. Clin. Transl. Oncol..

[262] Melegh Z., Oltean S. (2019). Targeting Angiogenesis in Prostate Cancer. Int. J. Mol. Sci..

[263] Eisermann K., Fraizer G. (2017). The Androgen Receptor of VEGF: Mechanism of Androgen-Regulated Angiogenesis in Prostate Cancer. Cancers (Basel).

[264] Bai W., Zhang W., Hu B. (2018). Vascular endothelial growth factor suppresses dendritic cells function of human prostate cancer. Onco. Targets Ther..

[265] Padró T., Ruiz S., Bieker R., Bürger H., Steins M., Kienast J., Büchner T., Berdel W.E., Mesters R.M. (2000). Increased angiogenesis in the bone marrow of patients with acute myeloid leukemia. Blood.

[266] Veiga J.P., Costa L.F., Sallan S.E., Nadler L.M., Cardoso A.A. (2006). Leukemia-stimulated bone marrow endothelium promotes leukemia cell survival. Exp. Hematol..

[267] Weidenaar A.C., ter Elst A., Koopmans-Klein G., Rosati S., den Dunnen W.F., Meeuwsen-de Boer T., Kamps W.A., Vellenga E., de Bont E.S. (2011). High acute myeloid leukemia derived VEGFA levels are associated with a specific vascular morphology in the leukemic bone marrow. Cell Oncol..

[268] Newell L.F., Holtan S.G. (2017). Placental growth factor: What hematologists need to know. Blood Rev..

[269] Jabari M., Allahbakhshian Farsani M., Salari S., Hamidpour M., Amiri V., Mohammadi M.H. (2019). Hypoxia-Inducible Factor1-Α (HIF1α) and Vascular Endothelial Growth Factor-A (VEGF-A) Expression in De Novo AML Patients. Asian Pac. J. Cancer Prev..

[270] Blackburn L.M., Bender S., Brown S. (2019). Acute Leukemia: Diagnosis and Treatment. Semin. Oncol. Nurs..

[271] Tang Y.T., Jiang F., Guo L., Si M.Y., Jiao X.Y. (2013). Expression and significance of vascular endothelial growth factor A and C in leukemia central nervous system metastasis. Leuk. Res..

[272] Macpherson G.R., Hanson C.A., Thompson D.M., Perella C.M., Cmarik J.L., Ruscetti S.K. (2012). Retrovirus-transformed erythroleukemia cells induce central nervous system failure in a new syngeneic mouse model of meningeal leukemia. Leuk. Res..

[273] Münch V., Trentin L., Herzig J., Demir S., Seyfried F., Kraus J.M., Kestler H.A., Köhler R., Barth T.F.E., Te Kronnie G. (2017). Central nervous system involvement in acute lymphoblastic leukemia is mediated by vascular endothelial growth factor. Blood.

[274] El-Obeid A., Sunnuqrut N., Hussain A., Al-Hussein K., Gutiérrez M.I., Bhatia K. (2004). Immature B cell malignancies synthesize VEGF, VEGFR-1 (Flt-1) and VEGFR-2 (KDR). Leuk. Res..

[275] Diffner E., Gauffin F., Anagnostaki L., Nordgren A., Gustafsson B., Sander B., Gustafsson B., Persson J.L. (2009). Expression of VEGF and VEGF receptors in childhood precursor B-cell acute lymphoblastic leukemia evaluated by immunohistochemistry. J. Pediatr. Hematol. Oncol..

[276] Padró T., Bieker R., Ruiz S., Steins M., Retzlaff S., Bürger H., Büchner T., Kessler T., Herrera F., Kienast J. (2002). Overexpression of vascular endothelial growth factor (VEGF) and its cellular receptor KDR (VEGFR-2) in the bone marrow of patients with acute myeloid leukemia. Leukemia.

[277] Dias S., Hattori K., Zhu Z., Heissig B., Choy M., Lane W., Wu Y., Chadburn A., Hyjek E., Gill M. (2000). Autocrine stimulation of VEGFR-2 activates human leukemic cell growth and migration. J. Clin. Invest..

[278] Casalou C., Fragoso R., Nunes J.F., Dias S. (2007). VEGF/PLGF induces leukemia cell migration via P38/ERK1/2 kinase pathway, resulting in Rho GTPases activation and caveolae formation. Leukemia.

[279] List A.F., Glinsmann-Gibson B., Stadheim C., Meuillet E.J., Bellamy W., Powis G. (2004). Vascular endothelial growth factor receptor-1 and receptor-2 initiate a phosphatidylinositide 3-kinase-dependent clonogenic response in acute myeloid leukemia cells. Exp. Hematol..

[280] Wang L., Zhang W., Ding Y., Xiu B., Li P., Dong Y., Zhu Q., Liang A. (2015). Up-regulation of VEGF and its receptor in refractory leukemia cells. Int. J. Clin. Exp. Pathol..

[281] Fragoso R., Pereira T., Wu Y., Zhu Z., Cabeçadas J., Dias S. (2006). VEGFR-1 (FLT-1) activation modulates acute lymphoblastic leukemia localization and survival within the bone marrow, determining the onset of extramedullary disease. Blood.

[282] Schmidt T., Kharabi Masouleh B., Loges S., Cauwenberghs S., Fraisl P., Maes C., Jonckx B., De Keersmaecker K., Kleppe M., Tjwa M. (2011). Loss or inhibition of stromal-derived PlGF prolongs survival of mice with imatinib-resistant Bcr-Abl1(+) leukemia. Cancer Cell.

[283] Ikai T., Miwa H., Shikami M., Hiramatsu A., Tajima E., Yamamoto H., Imai N., Hattori A., Nishii K., Miura K. (2005). Placenta growth factor stimulates the growth of Philadelphia chromosome positive acute lymphoblastic leukemia cells by both autocrine and paracrine pathways. Eur. J. Haematol..

[284] Karar J., Maity A. (2011). PI3K/AKT/mTOR Pathway in Angiogenesis. Front. Mol. Neurosci..

[285] Pinto M.P., Owen G.I., Retamal I., Garrido M. (2017). Angiogenesis inhibitors in early development for gastric cancer. Expert Opin. Investig. Drugs.

[286] Chen Z., Yang H., Li Z., Xia Q., Nie Y. (2019). Temsirolimus as a dual inhibitor of retinoblastoma and angiogenesis via targeting mTOR signalling. Biochem. Biophys. Res. Commun..

[287] Wu Y., Zhong Z., Huber J., Bassi R., Finnerty B., Corcoran E., Li H., Navarro E., Balderes P., Jimenez X. (2006). Anti-Vascular Endothelial Growth Factor Receptor-1 Antagonist Antibody as a Therapeutic Agent for Cancer. Clin. Cancer Res..

[288] Sawano A., Iwai S., Sakurai Y., Ito M., Shitara K., Nakahata T., Shibuya M. (2001). Flt-1, vascular endothelial growth factor receptor 1, is a novel cell surface marker for the lineage of monocyte-macrophages in humans. Blood.

[289] Imai N., Miwa H., Shikami M., Suganuma K., Gotoh M., Hiramatsu A., Wakabayashi M., Watarai M., Hanamura I., Imamura A. (2009). Growth inhibition of AML cells with specific chromosome abnormalities by monoclonal antibodies to receptors for vascular endothelial growth factor. Leuk. Res..

[290] Nielsen D.L., Sengeløv L. (2012). Inhibition of placenta growth factor with TB-403: A novel antiangiogenic cancer therapy. Expert Opin. Biol. Ther..

[291] Rizzo C., Yin Yin X., Packman K., Higgins B., Schnueriger A., Thomas M., Weidner M., Heimbrook D., Simcox M.E.C. (2011). Abstract 1370: RO5323441, a humanized monoclonal antibody against the placenta growth factor, blocks PlGF-induced VEGFR-1 phosphorylation in vitro and tumor growth in vivo. Cancer Res..

[292] Fischer C., Jonckx B., Mazzone M., Zacchigna S., Loges S., Pattarini L., Chorianopoulos E., Liesenborghs L., Koch M., De Mol M. (2007). Anti-PlGF inhibits growth of VEGF(R)-inhibitor-resistant tumors without affecting healthy vessels. Cell.

[293] Vahdat L.T., Layman R., Yardley D.A., Gradisha W., Salkeni M.A., Joy A., Garcia A.A., Ward P., Khatcheressian J., Sparano J. (2017). Randomized Phase II Study of Ramucirumab or Icrucumab in Combination With Capecitabine in Patients With Previously Treated Locally Advanced or Metastatic Breast Cancer. Oncologist.

[294] Petrylak D.P., Tagawa S.T., Kohli M., Eisen A., Canil C., Sridhar S.S., Spira A., Yu E.Y., Burke J.M., Shaffer D. (2016). Docetaxel As Monotherapy or Combined With Ramucirumab or Icrucumab in Second-Line Treatment for Locally Advanced or Metastatic Urothelial Carcinoma: An Open-Label, Three-Arm, Randomized Controlled Phase II Trial. J. Clin. Oncol..

[295] Moore M., Gill S., Asmis T., Berry S., Burkes R., Zbuk K., Alcindor T., Jeyakumar A., Chan T., Rao S. (2016). Randomized phase II study of modified FOLFOX-6 in combination with ramucirumab or icrucumab as second-line therapy in patients with metastatic colorectal cancer after disease progression on first-line irinotecan-based therapy. Ann. Oncol..

[296] Lassen U., Chinot O.L., McBain C., Mau-Sørensen M., Larsen V.A., Barrie M., Roth P., Krieter O., Wang K., Habben K. (2015). Phase 1 dose-escalation study of the antiplacental growth factor monoclonal antibody RO5323441 combined with bevacizumab in patients with recurrent glioblastoma. Neuro. Oncol..

[297] Martinsson-Niskanen T., Riisbro R., Larsson L., Winstedt L., Stenberg Y., Pakola S., Stassen J.M., Glazer S. (2011). Monoclonal antibody TB-403: A first-in-human, Phase I, double-blind, dose escalation study directed against placental growth factor in healthy male subjects. Clin. Ther..

[298] Lassen U., Nielsen D.L., Sørensen M., Winstedt L., Niskanen T., Stenberg Y., Pakola S., Stassen J.M., Glazer S. (2012). A phase I, dose-escalation study of TB-403, a monoclonal antibody directed against PlGF, in patients with advanced solid tumours. Br. J. Cancer.

[299] Wedge S.R., Kendrew J., Hennequin L.F., Valentine P.J., Barry S.T., Brave S.R., Smith N.R., James N.H., Dukes M., Curwen J.O. (2005). AZD2171: A highly potent, orally bioavailable, vascular endothelial growth factor receptor-2 tyrosine kinase inhibitor for the treatment of cancer. Cancer Res..

[300] Shirley M. (2018). Fruquintinib: First Global Approval. Drugs.

[301] Roskoski R. (2020). The role of fibroblast growth factor receptor (FGFR) protein-tyrosine kinase inhibitors in the treatment of cancers including those of the urinary bladder. Pharmacol Res..

[302] Du W., Huang H., Sorrelle N., Brekken R.A. (2018). Sitravatinib potentiates immune checkpoint blockade in refractory cancer models. JCI Insight.

[303] Li X., Li Y., Lu W., Chen M., Ye W., Zhang D. (2019). The Tumor Vessel Targeting Strategy: A Double-Edged Sword in Tumor Metastasis. Cells.

